# Determining Activity Patterns and Home Range of Wild Bats Using a Proximity Biologging System Based on the Internet of Things (IoT)

**DOI:** 10.1002/ece3.73604

**Published:** 2026-05-11

**Authors:** Jesús R. Hernández‐Montero, Janis Maximilian Wolf, Fernanda Chávez, Frieder Mayer, Gerald Kerth

**Affiliations:** ^1^ Zoological Institute and Museum, Applied Zoology and Nature Conservation Greifswald University Greifswald Germany; ^2^ Museum für Naturkunde Leibniz‐Institut für Evolutions Und Biodiversitätsforschung Berlin Germany

**Keywords:** bats, core area, home range, site fidelity, utilization distribution

## Abstract

Understanding spatial patterns in small, elusive species is critical for behavioral ecology and conservation, yet traditional tracking methods often face logistical constraints. We evaluated the utility of an Internet of Things (IoT)‐based proximity biologging system as an automated tool for determining the activity patterns and utilization distributions (UDs) of small, free‐ranging animals. We monitored female Bechstein's bats ( *Myotis bechsteinii* ) using a grid of 65 logging stations over two breeding seasons. Individual UDs were estimated using proximity data via autocorrelated kernel density estimation. We analyzed kinship‐driven space‐sharing and site fidelity through spatial overlap metrics. To validate system accuracy, we conducted a human‐mediated field simulation for comparing proximity against GPS‐derived UDs. Female bats exhibited individualized home ranges; however, mother‐daughter pairs showed significantly higher overlap than non‐related pairs. Repeatedly tagged individuals showed site fidelity. These results converge with previous patterns reported using VHF data. Field simulations demonstrated 88% (95% AKDE—home range) and 78% (50% AKDE—core area) of spatial congruence between proximity‐based and GPS‐derived UDs, confirming high locational accuracy within the detection grid. While the grid‐based approach limits UD estimations to the monitored area, the IoT proximity system provides a reliable and automated alternative for studying fine‐scale activity patterns. Our findings highlight the potential for this technology to identify activity hotspots and spatial dynamics in conservation‐priority habitats of species with relatively small home ranges and high site fidelity, such as the Bechstein's bat.

## Graphical abstract

Proximity data, traditionally used for inferring social networks, can inform utilization distribution at a fine scale. Using a sampling grid approach, it is possible to identify activity hotspots and spatial dynamics in conservation‐priority habitats.

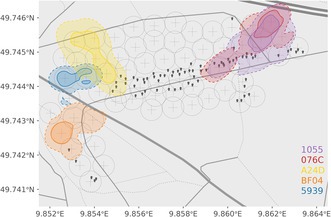

## 1 Introduction

Understanding how animals use space is fundamental to both behavioral ecology and conservation biology. One of the main approaches to achieve this understanding is based on the estimation of the home range, which represents the spatial extent an individual animal uses over time (Powell and Mitchell 2012). Home range estimations provide insights into species' movement ecology, site fidelity, resource preferences, and social behavior (Harris et al. 1990; Powell 2000). There are several analytical methods to estimate home ranges, with the minimum convex polygon (MCP) and kernel density estimation (KDE) among the most used by ecologists (Laver and Kelly 2008; Signer and Fieberg 2021).

Regardless of the method used to estimate home ranges, location data is the primary input for their calculation. Early works addressing home ranges in mammal species used very high frequency (VHF) telemetry to obtain location data (Harris et al. 1990). Although VHF telemetry studies have yielded insights into the home range of species with cryptic lifestyles, this method requires intensive and extensive field efforts to collect data and is prone to sampling bias (Cypher et al. 2014). However, technological advances have led to the miniaturization of consumer electronics, enabling the development of new, lightweight animal‐borne biologging devices that require little or no human mediation for data collection (Panicker et al. 2019; Wilmers et al. 2015). These new devices made it possible to track a wider variety of species, particularly small ones such as birds, rodents, and bats, and to collect large amounts of data (Ripperger et al. 2016; Wild, Wikelski, et al. 2023).

Most of the new technologies use wireless communication protocols such as Long Range (LoRa), Wi‐Fi, and Bluetooth low energy (BLE) that enable device communication within a local network known as the Internet of Things (IoT) (Wild, Van Schalkwyk, et al. 2023). Intra‐device communication at mid‐ or short‐range distances enables to infer social interactions (i.e., encounters) between tagged animals (Camal and Aksanli 2020; Davidson et al. 2025; Kirkpatrick et al. 2021). Such biologging systems capable of recording tag‐to‐tag encounters are known under the general term of proximity loggers (Krause et al. 2013). Proximity loggers, coupled with social network analysis, has proven to be a valuable tool in behavioral ecology (Ripperger, Günther, et al. 2019), sociobiology (Marsh et al. 2011; Ripperger, Carter, et al. 2019) and epidemiological research (Böhm et al. 2009; Ji et al. 2005) as they increase the quantity and quality of social network data (Ryder et al. 2012).

While proximity loggers have been mainly used to study animal interactions (Davidson et al. 2025), they can also be useful to infer animals' space use by analyzing contacts between tagged individuals and fixed monitoring locations. This is particularly useful when biologgers lack a Global Positioning System (GPS) component, or in complex environments, such as very dense forest vegetation or underground habitats, where GPS use is impractical due to poor satellite reception (Panicker et al. 2019; Ripperger et al. 2020). Data retrieval from proximity loggers is carried out wirelessly at gateways placed on the field without the necessity of recapturing the individual, unlike store‐on‐board GPS tags; therefore minimizing the risk of data loss (Huels et al. 2025).

Spatial analysis of animals tagged with proximity loggers requires the deployment of a detection grid consisting of receiver devices. For example, Ripperger et al. (2020) reconstructed the flight trajectories of free‐ranging mouse‐eared bats ( *Myotis myotis* ) by analyzing the angle‐of‐arrival of signals received by 17 logging stations distributed over a monitored area of approximately 1.5 ha. Thus, proximity loggers have the potential to inform analyzes of animal space use. However, to our knowledge, no studies have estimated home ranges using proximity data between tagged animals and receiver stations. In this field study, we tagged free‐ranging female Bechstein's bats ( *Myotis bechsteinii* ) with proximity loggers and monitored their space use by deploying a detection grid of logging stations within their core roosting area located in a deciduous forest in Germany.

Two behavioral characteristics of female Bechstein's bats make them suitable for being monitored with a proximity biologging system. First, they are specialized understory gleaning insectivorous bats. Characterized by short, broad wings and low wing loading, this species is capable of slow, maneuverable flight (Norberg and Rayner 1987). These morphological traits allow them to forage within closed forest and capture prey directly from surfaces (Güttinger and Burkhard 2013). Consequently, their foraging flight is typically restricted to low altitudes within the forest interior (Siemers and Swift 2006), increasing their detection probability by ground‐based logging stations. Second, they exhibit high foraging site fidelity, typically hunting close to their day‐roost within an average distance (mean ± SD) of 147 ± 159 m (Kerth, Wagner, and König 2001; Kerth and Morf 2004). This restricted spatial range makes this species highly feasible to deploy a high density detection grid within their core roosting area to capture individuals' spatial patterns.

We estimated utilization distributions (UDs), that is, the relative frequency distributions in a 2D‐plane of an animal's locations over time (Van Winkle 1975), of free‐ranging female Bechstein's bats ( *Myotis bechsteinii* ) within a maternity colony. We then determined space sharing (i.e., UD overlap), and site fidelity of repeatedly tagged individuals in different periods. Based on previous VHF telemetry studies determining the home range of Bechstein's bats, we expect to observe (1) individualized foraging areas; (2) a higher degree of overlap in the UDs of kin‐related (i.e., mother‐daughter pairs) individuals compared to unrelated colony members, and (3) site fidelity of repeatedly tagged individuals to their foraging areas (Kerth, Wagner, and König 2001; Melber et al. 2013).

Additionally, we evaluated the accuracy of the Bluetooth low energy proximity system to determine UDs, through a human‐mediated field simulation. During the simulation, researchers simultaneously collected proximity and GPS location data to determine if proximity‐based UDs reflect the spatial patterns observed in GPS‐derived data. Given the higher positional accuracy of GPS data, due to good satellite reception in our study site, we used the UDs derived from it as a reference. We expected a high overlap probability between the UDs derived from both methods indicating the reliability of proximity data. In combination, the results of this field study provide insights into whether proximity data are reliable to address space use of free‐ranging animals using an automated approach.

## 2 Methods

### 2.1 Study Site and Species

Fieldwork took place in May and August of 2024 and 2025 in a deciduous forest located close to the city of Würzburg, Germany. This forest is home to a maternity colony (GB2) of Bechstein's bats ( *Myotis bechsteinii* ) which has been subject to continuous monitoring since 1996 (Kerth et al. 2002). All adult females are marked within their first year of life with a subcutaneous radio frequency identification (RFID) transponder (Trovan ID‐100C, United Kingdom). Additionally, at the first capture, a wing‐membrane biopsy punch is collected to obtain a DNA sample for genetic analysis to construct pedigrees based on 13 polymorphic microsatellite loci (Mundinger et al. 2022).

**FIGURE 1:**
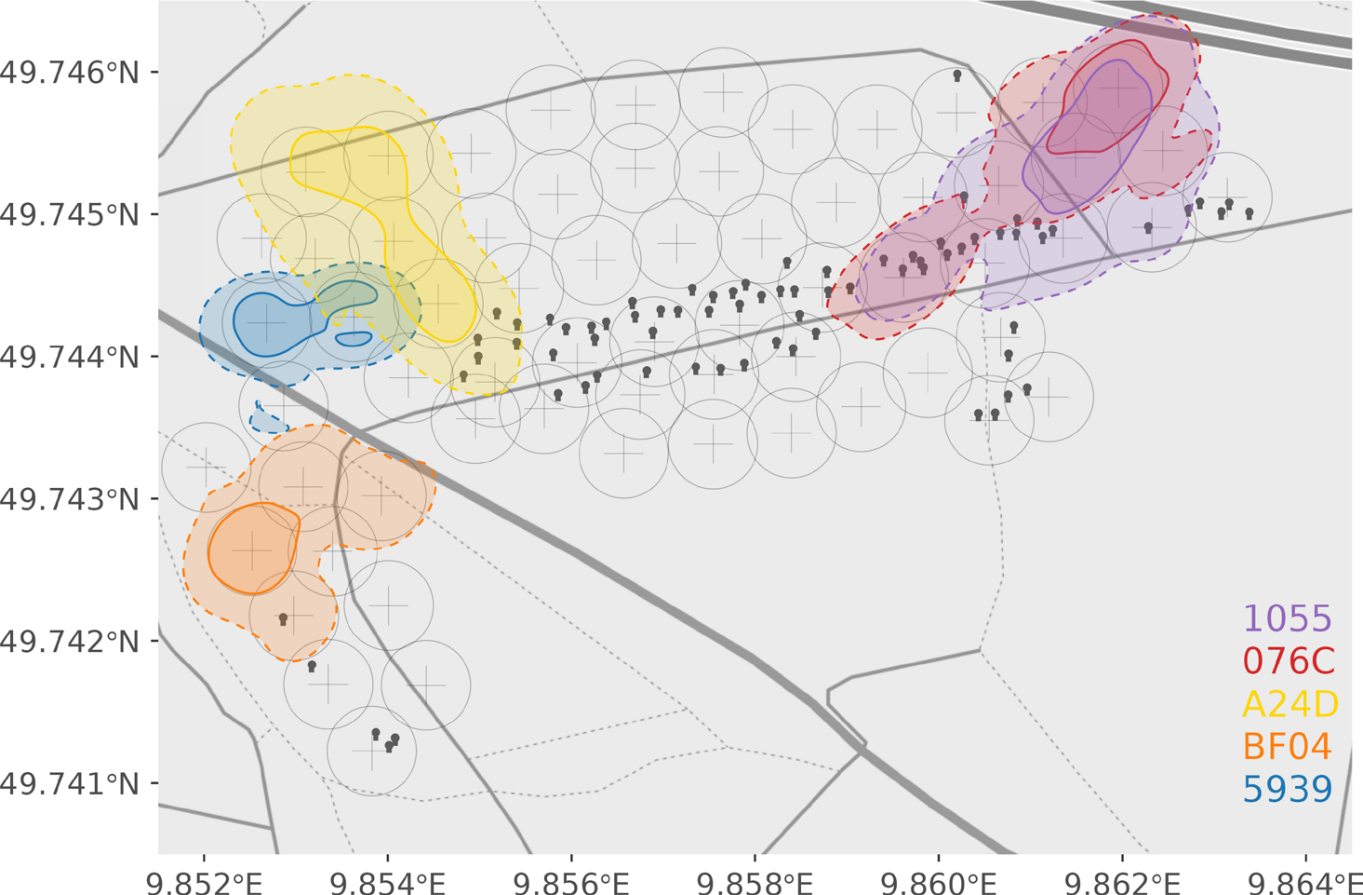
Home range (AKDE 95%, dashed lines) and core areas (AKDE 50%, solid lines) of five free ranging Bechstein's bats represented in different color shades. Bats were marked in August 2024. Individuals “076C” and “1055” are a mother‐daughter pair. Crosses indicate the location of stationary loggers, circles around them indicate the 35 m radius corresponding to the detection range. Black symbols represent bat boxes.

In 2024, there were 72 adult females while in 2025 there were 50 adult females in GB2. At the study site (*ca*. 0.4 km^2^), bats mainly roost in bat boxes (type 2FN, Schwegler, Germany) and occasionally in tree cavities. We daily inspected the bat boxes to identify day roosts and monitored them using RFID loggers. During the study period, there were 75 bat boxes distributed across the roosting area (Figure 1).

### 2.2 Proximity Monitoring

Activity monitoring outside day roost was carried out using an IoT proximity biologging system, ProxLogs (IoSA, Antwerp, Belgium). This system uses Bluetooth Low Energy (BLE) transmission to register contacts between mobile and stationary components (Kirkpatrick et al. 2021). The system has four components: (1) Micro loggers (ML), (2) Stationary loggers (SL), (3) a gateway, and (4) a mobile phone application. The MLs were powered by 13 mAh LiPo batteries, and were wrapped in a nitrile fingertip glove for a total mass of ~1.1 g. The SLs were powered by 800 mAh LiPo batteries, and we deployed them at fixed locations within the colony's roosting area to build a detection grid (see below). Both SLs and MLs have a unique numerical identity to facilitate data management. The gateway is dedicated for timestamp synchronization and remote data retrieval from ML and SL. The Mobile phone application facilitates the wireless adjustment of the sampling behavior of components (e.g., sample rate, sampling schedule, transmission behavior and transmission power) and components monitoring (e.g., memory and battery status).

Based on previous knowledge from VHF telemetry conducted in the same study site (Melber et al. 2013), we built a detection grid using 65 SLs covering the main roosting area and foraging habitat in the GB2 colony (Figure 1). We placed each SL in a water‐resistant electric box and mounted them on a PVC pole at a height of 2 m above the ground. The distance between neighboring SLs was 65 m, based on a detection range of 35 m, allowing a certain overlap in the detection range between two neighboring SLs. Based on preliminary equipment calibration in field conditions, a ML (with boosted transmission power) at a height of 1.3 m above the ground can be detected by a SL at a range of up to 65 m. However, because the number of recorded beacons drops after 40 m, a conservative separation between SLs was decided upon to ensure reliable ML detection (see Appendix S2 “Calibration – SL detection range”). Using a mobile‐phone GPS application (OsmAnd v.5.0.5), we recorded the geographic coordinates of the SLs and then estimated the distance between SLs, which we subsequently corroborated with a measuring tape in the field. The exact location of each SL varied slightly from one year to another with a mean ± SD distance difference (2024 vs 2025) of 11.7 ± 13.41 m.

We configured the SLs with a sampling schedule encompassing the period of nocturnal activity of bats between 21:00 and 05:00. The sample rate was set to 2 s for both SL and ML with a boosted transmission power to ensure a longer detection range between components and to facilitate data retrieval over greater distances. All SLs were set in “hidden mode”, that is, they were not transmitting beacons but only recording those from the MLs. In this way, we avoided recording uninformative encounters between SLs. Therefore, the data only include records of MLs detected by each SL. The internal memory of a SL allows to record up to 4000 encounters. Once the data are retrieved using a gateway, the memory is automatically wiped. To avoid memory saturation, we installed a gateway near current day roosts, as we expected activity to be concentrated in those areas. We installed a gateway in the field and configured it to download data once an SL reached 300 records. We retrieved the data of the remaining SL, that were out of the detection range of the installed field gateway, using a second gateway with a download threshold of one record.

### 2.3 Data Management

The data retrieved by each SL are saved in CSV format. Each file is named according to the SL's identity and contains the following data: receiver ID (SL identity), sender ID (ML identity), timestamp of the meeting, and signal strength of the record (RSSI). We merged all the CSV files from the SLs into a single database and sorted it by the timestamp of the records. Then, we complemented the database with the geographic coordinates of each record (i.e., SL location). We filtered out records with a RSSI equal or lower than −90 dB as they likely represent noise given its weak signal strength. We then sliced the database into data sets corresponding to each ML and used these data sets to build maps projecting individual utilization distributions (i.e., home range and core area).

### 2.4 Estimation of Utilization Distributions

The records of ML by each SL were accounted as fixes, and they were used to estimate utilization distribution maps using R‐programming language (R Core Team 2023). Since each fix is linked to the location of a given SL, we randomly assigned the location of each fix according to its signal strength value within a certain area around the SL where the ML had been recorded. Since RSSI decreases with distance, records with an RSSI equal to or higher than −82 dB were placed within a 15‐m radius circle around the corresponding SL. Records with an RSSI lower than −82 dB were placed within a radius ranging from 15 to 35 m. We determined distance ranges based on previous outdoor tests of the equipment prior to its deployment. This random allocation falls within the detection range of the SL and avoids clumping fixes with identical locations prior to utilization distributions estimations.

We estimated utilization distributions (UD) using kernel density estimation. Since our sampling method is prone to spatial autocorrelation, given its high sampling rate and the grid approach followed, we use the autocorrelated kernel density estimation (AKDE; Hollins et al. 2025). This method accounts for the temporal autocorrelation of the location data, resulting in more accurate and unbiased UDs. The AKDE adjusts for the dependence between successive locations and does not require the selection of a smoothing parameter (Fleming et al. 2015). We calculated 95%‐AKDEs to determine individual home ranges, and 50%‐AKDEs as core areas (Samuel et al. 1985). Home range and core area size estimations via AKDE were computed using the R‐package Animal Movement Tools (Signer et al. 2019, 2024).

Since we used a detection grid, we investigated whether the estimated UD sizes were biased by the amount of time an individual spent within its detection range. For each bat, we calculated the detection percentage based on the number of minutes the bat was detected by at least one SL of the detection grid relative to the total expected minutes of activity used to estimate UD. Then, we performed a Spearman's rank correlation test to evaluate the relationship between bat detection percentage and estimated UD size.

### 2.5 Home Range and Core Area Overlap

After determining the UDs (i.e., home range and core area) of each individual, we calculated the pairwise overlap to address (1) pairwise space‐use sharing, (2) site fidelity of repeatedly tagged bats, and (3) differences between UDs derived from different biologging methods (ProxLogs vs GPS; see Section 2.9). Space‐use sharing of free ranging bats was determined between individuals monitored during the same period. To address site fidelity, we quantified the overlap of UDs calculated in different monitoring periods either within the same season (i.e., same year) or between different years. Finally, we compared the reliability of the UDs derived from our IoT proximity system with those derived from GPS data, using the latter as a reference.

We used two indices proposed by Fieberg and Kochanny (2005) to quantify overlap: the utilization distribution overlap index (UDOI) and Bhattacharyya's affinity (BA). Both indices are non‐directional functions of the joint distribution of two UDs. This means they provide a unique probabilistic value ranging between 0 (no overlap) and 1 (for 2 individuals with identical UD), based on the combined distribution of the two individuals' UDs. In the case of UDOI, values can be greater than 1 if the two individuals have a higher overlap relative to uniform space use, meaning that two individuals have high overlap and are both concentrated in the same high‐use areas. Following the recommendations of Fieberg and Kochanny (2005), we addressed space‐use sharing of bats with the UDOI index. While we addressed bats' site fidelity, and compared the UD derived from different monitoring methods with the BA index. We calculated overlapping indices using the R‐package Animal Movement Tools (AMT, Signer et al. 2024).

### 2.6 Bat Tagging and Monitoring

We tagged three batches of bats in 2024 and two batches in 2025 (see Table 1). Twenty‐three different individuals were tagged with a ML in 2024, and 10 in 2025. Four individuals were tagged more than once within the same year, but in different months (May and August), and nine individuals in different years (see Appendix S1). In both years, we carried out the monitoring with the IoT proximity system in May and August to avoid disturbing females that were in an advanced stage of pregnancy or lactating in June and July (Melber et al. 2013). Overall, we obtained proximity location data from 25 adult females.

**TABLE 1:** Individuals monitored in five different batches. The number of possible dyads and mother‐daughter pairs considers only the individuals monitored during more than one night (shown in parenthesis).

Batch	No. bats (with info)	Dyads	Mother‐daughter pairs	Start date	End date
May 24	8 (7)	21	0	2024‐05‐15	2024‐05‐17
August 24a	12 (11)	55	5	2024‐08‐12	2024‐08‐14
August 24b	6 (5)	10	1	2024‐05‐15	2024‐05‐17
May 25	8 (5)	10	2	2025‐05‐14	2025‐05‐16
August 25	6 (6)	15	1	2025‐08‐07	2025‐08‐09

We tagged adult females after capturing them by hand from their day roost during the morning. We identified each individual using an RFID hand‐reader, and recorded their body mass using an electronic scale with a precision of 0.01 g. Relatedness of tagged individuals was determined using pedigrees based on 13 polymorphic microsatellite loci (Mundinger et al. 2022). Dyads of tagged bats were then categorized as kin (i.e., mother‐daughter) and non‐related.

We wrapped MLs in a nitrile fingertip glove and glued it between the bats' scapulae using a skin adhesive (Sauer‐Hautkleber 50%, Manfred Sauer, Germany). We only tagged individuals with a body mass of at least 11 g for a tag to body mass ratio of less than 10%. While our tag‐to‐body mass ratio exceeds the recommended 5% threshold (O'Mara et al. 2014), several factors ensure avoiding a long‐lasting disturbance of tags. First, the skin adhesive degrades naturally, and tags were retrieved within 3–4 days. Second, there was a high individual recapture probability at day roosts. Third, the low wing loading of Bechstein's bats provides a higher theoretical load‐lifting capacity (Norberg and Rayner 1987). Furthermore, a recent study suggests that tag masses up to 10% are acceptable for short‐term studies (Kelling et al. 2024). Finally, the presence of non‐recapture individuals was confirmed through RIFD data from day roost monitoring. We recorded the body mass of individuals at the moment of tagging and at their closest first recapture. First recapture after tagging ranged from 3 to 18 days. We then quantify individuals' change in body mass (capture weight—first recapture weight) where positive values indicate weight lost. Tagged individuals lost on average (±SD) 0.40 ± 0.80 g (*n* = 18 recaptured individuals) while non‐tagged individuals gained weight −0.76 ± 0.74 g (*n* = 29). A temporary weight loss in this range has also been observed in other bat species tagged with different biologging devices (Kelling et al. 2024; Meierhofer et al. 2024).

Handling and tagging of the bats were conducted under permits for species protection (RUF‐55.1.2‐8646.2‐5‐44‐2) and animal welfare (RUF‐55.2.2‐2532‐2‐620‐7) issued by the government of Lower Franconia.

### 2.7 Space‐Use Sharing

We analyzed the degree of overlap of the UD among bats that provided at least two nights of data. To avoid overestimation of home ranges and core areas, we used the data collected 2 h after sunset and 3 h before sunrise. In this way, we ensured that UDs reflect individual activity outside their day roost by excluding activity close to it after leaving and before entering (Kerth, Weissmann, and König 2001). We assessed space‐use sharing by quantifying the overlap between different individuals tagged in the same batch using the UDOI index.

To evaluate whether dyadic spatial overlap in home range and core area was influenced by kinship (mother‐daughter vs. non‐related), we employed a Generalized Linear Mixed Model (GLMM) via the glmmTMB R‐package (Brooks et al. 2017). Kinship was classified as “mother‐daughter” pairs and “non‐related” if they were not mother‐daughter pairs. Non‐related pairs may include sisters and aunt‐niece pairs. The degree of overlap (UDOI) was modeled as a function of kinship, with dyad identity included as a random effect to account for the non‐independence of repeated pairwise measures. Mother‐daughter pairs were defined as the reference level. Given the continuous, non‐negative nature of the overlap data and the high frequency of zero values, we used a Tweedie error distribution with a log‐link function. Furthermore, to address heteroscedasticity identified through residual diagnostics, we specified a dispersion formula within the GLMM. This approach allowed for independent variance estimation for mother‐daughter and non‐related dyads, ensuring the calculation of robust standard errors for the fixed effects.

### 2.8 Site Fidelity

We assessed site fidelity for individuals repeatedly tagged across different periods using the Bhattacharyya's affinity index (BA; Fieberg and Kochanny 2005). We quantified overlap for bats tagged within the same breeding season (i.e., May and August; *n* = 4) and across different years (i.e., 2024 and 2025; *n* = 9). We reported the range and mean (±SD) of BA values for both within‐year and between‐year recaptures.

To evaluate changes in utilization distribution area size between the first and second monitoring periods, we pooled data from all individuals tagged repeatedly within or across years (*n* = 13, from 10 unique bats). We employed a Linear Mixed‐Effects Model (LMM) rather than a non‐parametric approach to better account for the non‐independence of data from individuals contributing multiple trials. The model was structured with the period of monitoring (first vs second) as a fixed effect and individual bat ID (RFID) as a random effect to control for repeated measures. Models were fitted using the lme4 R‐package (Bates et al. 2015), and *p*‐values were derived using Satterthwaite's degrees of freedom approximation via the lmerTest R‐package (Kuznetsova et al. 2017). Model diagnosis was done through visual inspections of residuals.

### 2.9 Monitoring Methods Comparison

Finally, we addressed the reliability of the estimated UDs based on proximity data by comparing its size and overlap with those derived from GPS data. We collected data simultaneously from MLs and GPSs (OsmAnd v.5.0.5) during seven tracks within the detection grid that we had set up in the core roosting area (see Appendix S2). Three tracks were conducted in May and four in August 2025. During each track, a person carried a ML and a GPS. Mobile loggers were mounted on 2 m high poles and were set with boosted transmission power at a sample rate of 2 s; the 65 SLs from the detection grid were set in hidden mode. Each person tracked a 45‐min walk with a GPS set to take a waypoint every 2 s to match the sample rate of the MLs. Waypoints taken during the track of the walks were accounted as fixes for calculating UDs. Every person intentionally used a specific area of the forest to recreate an individual home range and core area. All participants started and ended at the same point, simulating the emergence and return of bats to a daytime roost. However, to avoid individuals' home ranges and core areas overlapping close to the starting and ending points, we discarded the first and last 10 min of the tracks. The UDs were calculated based on AKDEs at 95% (home range) and 50% (core area) and were compared pairwise between monitoring methods (ProxLogs vs GPS). The areas of the UDs were compared using a two‐sided paired *t*‐test after addressing normality and homoscedasticity. We addressed the overlap between areas derived from the two monitoring methods using the BA index (Fieberg and Kochanny 2005).

## 3 Results

### 3.1 Home Range and Core Areas of Bechstein's Bats

Of the 25 individuals tagged, 21 provided data for more than one night. Tags were operating for (mean ± SD) 2.55 ± 0.50 nights resulting in 1517 ± 1048 fixes per bat that could be used for UD overlap analysis. The estimated home range (AKDE 95%) areas had a mean (± SD) of 3.11 ± 1.57 ha ranging from 0.98 to 7.07 ha (*n* = 21). Core areas (AKDE 50%) mean size was 0.58 ± 0.36 ha ranging from 0.14 to 1.83 ha (*n* = 21).

The mean (± SD) percentage of time that bats were recorded inside the detection grid was 42.94% ± 16.39% ranging from 17% to 78%. Despite this variance, the Spearman correlation tests revealed no significant relationship between bats' percentage of detection and their home range (*r* = −0.08, *p* = 0.63) and core area size (*r* = 0.11, *p* = 0.51).

### 3.2 UD Overlap—Space Sharing

We addressed space‐sharing across 103 different dyads, 96 of which were non‐related according to our definition, and seven were mother‐daughter pairs. Some dyads were sampled more than once with six non‐related dyads sampled twice, two mother‐daughter pairs twice and one mother‐daughter pair in three different periods. Utilization distribution overlap of individuals marked in the same period (i.e., batch) was calculated with the UDOI statistic. Irrespective of kinship, bats show a home range overlap (mean ± SD, *n* = 111) of 0.30 ± 0.52 ranging from 0 to 2.19. Core areas have a lower overlap with 0.09 ± 0.21 ranging from 0 to 0.84. Figure 1 shows the projection of the UD of a batch of bats marked in August 2024, UD projections of all sampled batches are shown in Appendix S1.

### 3.3 Overlapping Degree by Kinship

**FIGURE 2:**
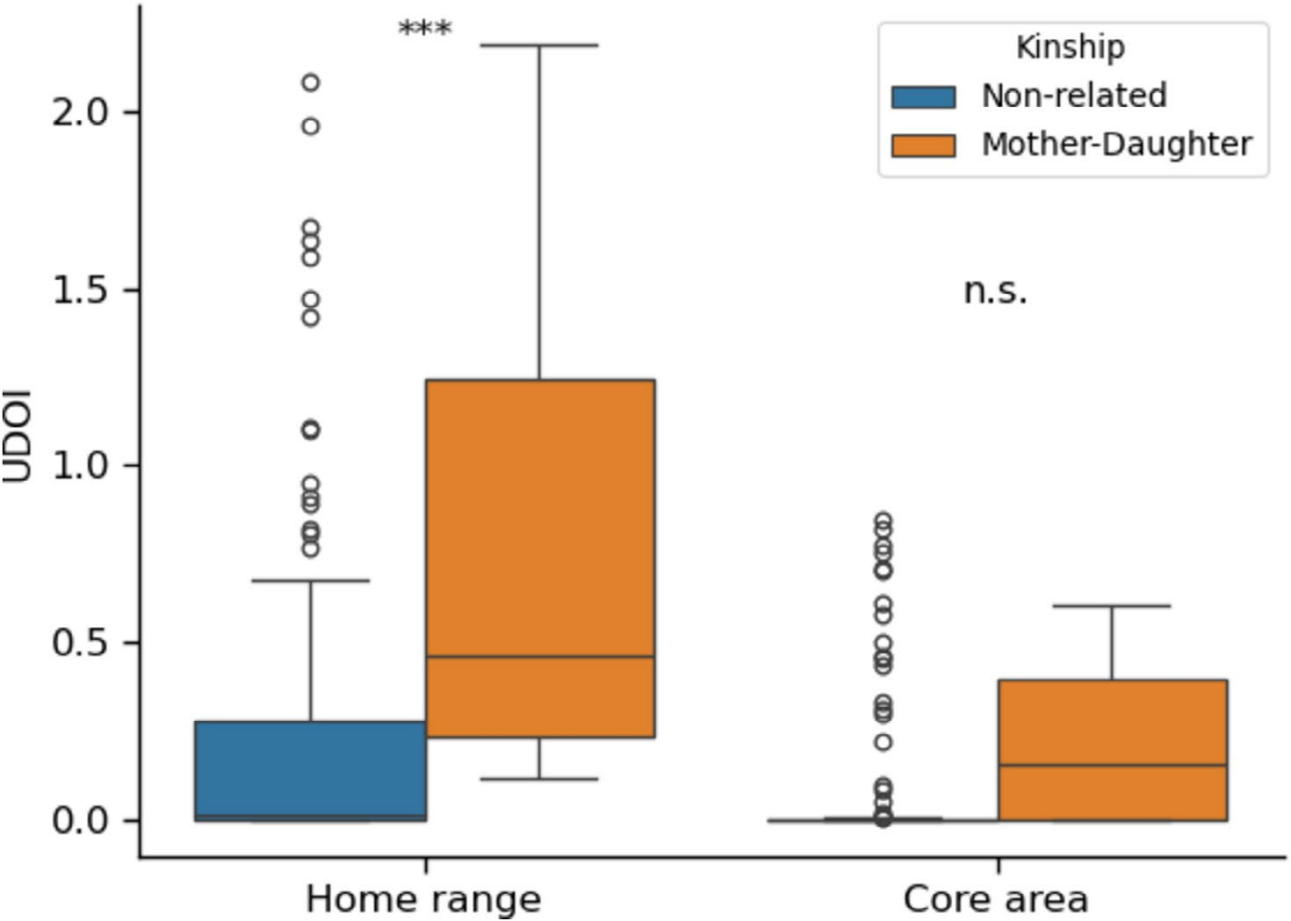
Box plot of the overlapping degree (UDOI) in home range (AKDE 95%) and core area (AKDE 50%) by kinship. Significant values derived from GLMM models comparing the overlap between mother‐daughter pairs (*n* = 7) and other pair types (*n* = 96).

Our GLMM model revealed that kinship had a significant effect on both the magnitude and variability of dyadic home range overlap (ß = −1.03 ± 0.45 SE, *z* = −2.29, *p* = 0.02) with non‐related pairs exhibiting approximately 65% less overlap compared to mother‐daughter pairs (exp (−1.03) ≈0.35). Furthermore, the dispersion model indicated significant heteroscedasticity between groups (*z* = 2.94, *p* = 0.003). Non‐related pairs displayed significantly greater variability in their overlapping degree compared to mother‐daughter pairs (Dispersion Estimate = 2.01 ± 0.68 SE, Figure 2).

In the case of core area overlap, we found that kinship did not significantly influence the overlapping degree (ß = −0.86 ± 0.63 SE, *z* = −1.36, *p* = 0.174). However, the dispersion model revealed a significant difference in variability with non‐related pairs exhibiting higher variance than mother‐daughter pairs (*z* = 2.51, *p* = 0.012, Figure 2).

### 3.4 Site Fidelity

**FIGURE 3:**
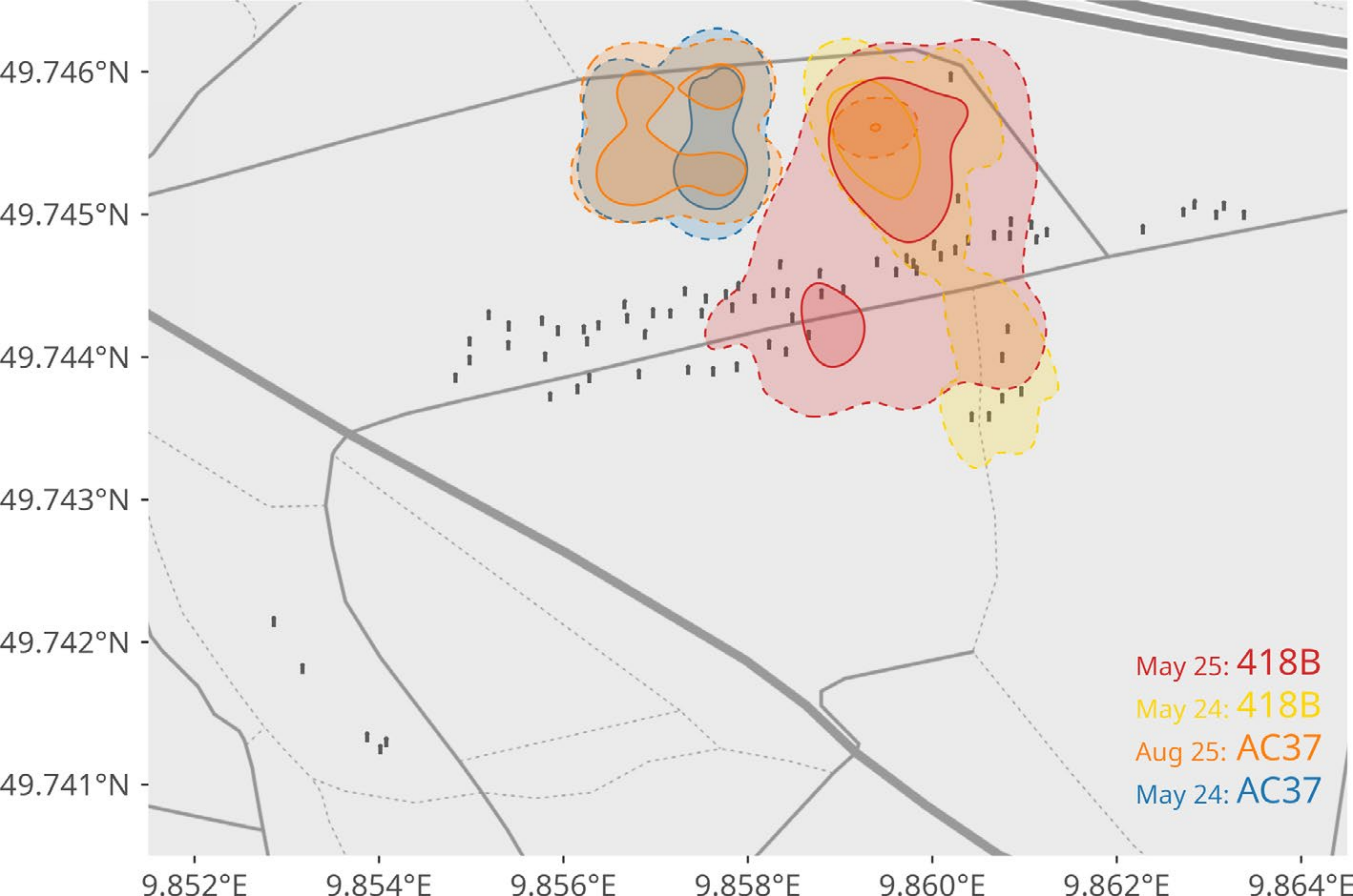
Inter‐annual site fidelity of the home range (AKDE 95%, dashed lines) and core areas (AKDE 50%, solid lines) of two individuals (“418B” and “AC37”) tagged in 2024 and 2025. Bats showed a higher fidelity to their home range compared to their core areas.

According to the BA index, the four bats tagged twice in the same breeding season exhibit a home range overlap (mean ± SD) of 0.58 ± 0.23 and a core area overlap of 0.35 ± 0.30. In the case of site fidelity between years, the nine individuals marked in 2024 and 2025 also showed similar home range (0.57 ± 0.23) and core area (0.27 ± 0.26) overlap (see Figure 3).

When comparing the area size between the first and the second period of monitoring of all repeatedly tagged bats, we found no significant difference for both home range (LMM: ß = −0.17 ± 0.53 SE, *t* = −0.32, *p* = 0.757) and core area (ß = −0.03 ± 0.17, *t* = −0.17, *p* = 0.86). While the home range model showed moderate consistency within individuals (RFID‐identity accounted for ~30% of total variance), the core area model exhibited negligible individual‐level variance.

### 3.5 Methods Comparison: Proximity vs. GPS

**FIGURE 4:**
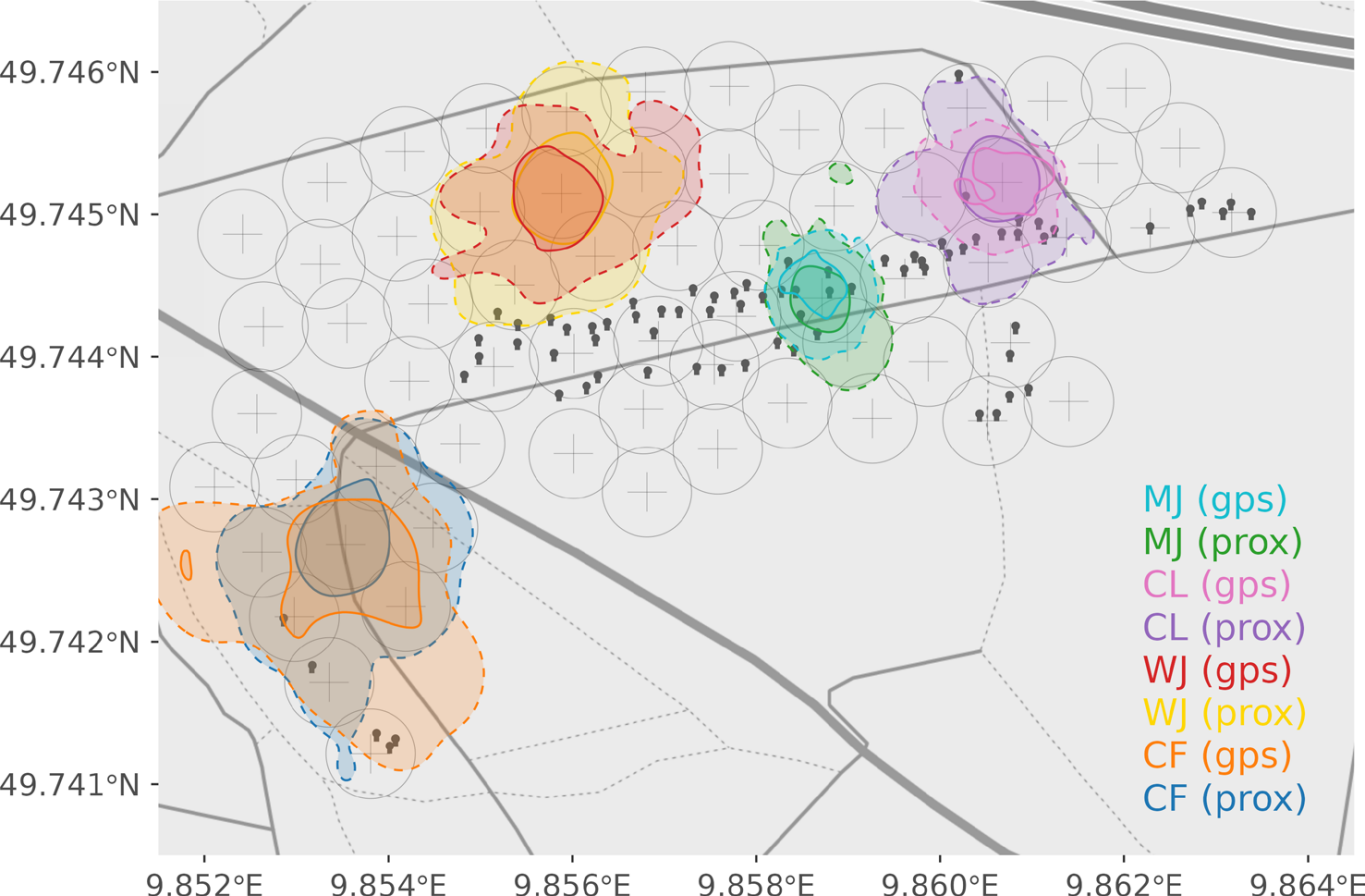
Home range (AKDE 95%, dashed lines) and core areas (AKDE 50%, solid lines) calculated from location data simultaneously collected with proximity loggers (“prox”) and GPS (“gps”) data during four walks. Crosses indicate the location of stationary loggers, circles around them indicate the 35 m radius corresponding to the detection range. Black symbols represent bat boxes.

We obtained a total of 3710 and 3294 fixes, using proximity loggers (ProxLogs) and GPS respectively, for calculating the utilization distributions of seven tracks within the monitored area. The UDs calculated with ProxLogs were larger except for one track (see “CF” in Figure 4). Although the home range size calculated with ProxLogs was on average (mean ± SD) 48.22% ± 44.81% larger, there was no significant difference in the area returned by both methods (*t* = −1.69, df = 6, *p* = 0.14, *n* = 7). Home ranges derived from GPS and ProxLogs overlapped on average 0.88 ± 0.02 according to the BA index (Table 2). Similarly, core areas calculated with ProxLogs were 23.45% ± 45.95% larger than those calculated with GPS. However, there was no significant difference between the areas returned by both methods (*t* = −0.67, df = 6, *p* = 0.52, *n* = 7). The overlapping degree of the core areas was on average 0.78 ± 0.06 according to the BA index (Table 2).

**TABLE 2:** Utilization distribution areas (ha) calculated with proximity (Prox.) and GPS data. The table shows the percentage of overestimation (Overest. %) calculated with proximity data in comparison to GPS data. Overlap degree is based on the Bhattacharyya's affinity index.

Ind	Home range				Core area			
	Prox. (ha)	GPS (ha)	Overest %	Overlap	Prox. (ha)	GPS (ha)	Overest %	Overlap
JW	2.93	2.08	40.87	0.88	0.72	0.46	56.52	0.80
LC	2.63	1.45	81.38	0.88	0.60	0.42	42.86	0.79
JM	3.36	1.66	102.41	0.84	0.88	0.43	104.65	0.77
CF	3.05	4.09	−25.43	0.86	0.50	0.92	−45.65	0.79
WJ	2.70	2.16	25.00	0.89	0.50	0.42	19.05	0.86
CL	1.74	0.90	93.33	0.87	0.33	0.26	26.92	0.76
MJ	0.97	0.67	44.78	0.92	0.19	0.16	18.75	0.66

## 4 Discussion

We employed a proximity biologging system to estimate utilization distributions (UDs) of free‐ranging Bechstein's bats. By quantifying spatial overlap, we characterized kinship‐driven patterns of space‐sharing and individual site fidelity. Estimations accuracy was validated via human‐mediated field simulations. By collecting location data with proximity loggers and GPS simultaneously, we were able to contrast the size and spatial congruence of UDs derived from both monitoring methods. Although our monitoring and analytical techniques differ from those used in previous studies (Kerth, Wagner, and König 2001; Kerth and Melber 2009; Melber et al. 2013), we aim to determine whether our results converge into analogous spatial patterns reported for Bechstein's bats.

### 4.1 Utilization Distribution Areas

The number of fixes obtained from proximity data was considerably higher than those obtained using VHF over longer periods of monitoring. This is due to the high sample rate (2 s) of the BLE proximity system and its ability to track several individuals simultaneously. Previous studies in the same colony (GB2) using VHF achieved 137 ± 36 fixes per bat (*n* = 14) in 4.1 ± 1.3 nights (Kerth and Melber 2009). In our case we recorded 1516.5 ± 1047.79 fixes per bat in 2.55 ± 0.5 sampling nights.

Despite having an 11‐times higher number of fixes per bat, both home range and core areas were considerably smaller than those previously calculated from VHF fixes in the GB2 colony (Kerth and Melber 2009). The mean (±SD) home range area (AKDE 95%: 3.16 ± 1.58 ha) was three times smaller than those calculated using KDE 90% and two times smaller using MCP 95%. The mean core areas (AKDE 50%: 0.61 ± 0.36 ha) derived from proximity data were up to 3.6 times smaller than those calculated using KDE 50% (Kerth and Melber 2009; Melber et al. 2013).

The marked differences in UDs size can be rooted in the monitoring method, sampling schedule, and analytical method. Contrary to previous studies that calculated UDs using fixes collected during the whole night, we decided to use a subset of the data that clearly reflects the activity away from the day roosts. Therefore, we filter out potential activity around day roosts given the swarming behavior of bats at sunset and dawn (Kerth, Weissmann, and König 2001). However, differences remain the same even after estimating UDs using unfiltered proximity data (results not shown). Thus, the most likely reason behind the differences in UDs sizes previously reported is grounded in our grid monitoring approach.

The detection grid employed constraints the size of the calculated UDs because the bat activity outside the grid is not taken into account for its estimation. However, the non‐significant correlation between bats' percentage of detection inside the grid and the size of their UDs suggests that the UDs reflect the individual movement patterns within the colony's roosting area. Nevertheless, expanding the detection grid can provide spatial use information further away from the core colony area.

### 4.2 Space Use Sharing

The low overlap in both home range (UDOI mean ± SD: 0.29 ± 0.48) and core areas (0.09 ± 0.20) confirm highly individualized areas. This results is in accordance with Kerth, Wagner, and König (2001) who also found a low overlap in the home range (MCP 100%) of Bechstein's bats (mean ± SD overlap: 12.3% ± 24.4%).

When analyzing the home range overlapping degree by kinship, we found that mother‐daughter pairs overlap significantly more than non‐related pairs (*p* = 0.02). Contrary to home range, we found no differences in the overlapping degree of core areas between kinship groups (*p* = 0.174). For both UDs, the dispersion model indicated significant heteroscedasticity suggesting that mother‐daughter dyads maintain more consistent levels of home range and core area overlap than non‐related individuals.

Higher home range overlap between mother‐daughter pairs is in concordance with previous studies (Kerth, Wagner, and König 2001; Melber et al. 2013). However, we did not find a higher degree of core area overlap between kin‐related individuals as was found by Melber et al. (2013). This could be explained by the smaller size of the estimated areas in our study. The high degree of home range overlap likely reflects the maternal inheritance of foraging grounds, where young females are recruited into. However, the lower degree of overlap in core areas between mother‐daughter pairs may reflect fine‐scale resource partitioning among individuals. By maintaining separate core activity centres, mothers and daughters may minimize competition while benefiting from the shared knowledge of the broader forest habitat.

### 4.3 Site Fidelity

According to our LMM area analysis, all repeatedly tagged individuals showed a similar home range and core area size estimated in different periods. Interestingly, the identity of the individuals (RFID) accounted for approximately 30% of the total variance in the size of the home range, but it did not contribute to the variance of the core area size. This suggests that while total space use may be somewhat characteristic of an individual, the size of intensively used core areas is highly variable and may be driven by resource availability.

Home range overlap showed that all individuals exhibited a certain degree of overlap either if they were retagged in the same season (BA mean ± SD: 059 ± 0.23) or in different years (0.57 ± 0.25). Core areas overlapped to a lower degree compared to home ranges of bats monitored twice in the same season (0.35 ± 0.29) and in different years (0.28 ± 0.27). This suggests that bats exhibit a higher consistency in their home range location while the core area contained within it is more variable not only in its size but also in its location. These results are in accordance with Melber et al. (2013) who also observed a higher overlapping degree in the home range than in the core areas.

Analyzing spatial patterns using proximity data, our study converges on analogous spatial patterns of free‐ranging Bechstein's bats described using VHF data. Our results show that Bechstein's bats exhibit the following characteristics: (1) individualized UDs; (2) home range of mother‐daughter overlap in a higher degree than non‐related pairs; and (3) a high site fidelity within and between breeding seasons.

### 4.4 Comparisons Between Proximity and GPS‐Derived UDs

The home ranges (AKDE 95%) as well as the core areas (AKDE 50%) calculated with proximity data were in general larger than those calculated with GPS data. Utilization distribution areas were up to twice the size of those calculated using GPS data (Table 2). However, there was one exception: the home range and core area estimated with proximity data for individual “CF” were 25% and 46% smaller compared to those derived from GPS data (see Table 2 and Figure 4). This is explained because individual “CF” walked beyond the boundaries of the detection grid.

Differences in UDs area sizes can be explained due to our detection grid approach used with the proximity biologging system. Contrary to GPS data where each fix has a unique estimated location, fixes recorded within the detection grid have a fix location of a given stationary logger (SL) but the true location of the individual is not reflected. For further UD estimations, a location within the detection range of a logging station was randomly assigned within the SL's detection range. Consequently, proximity UD areas are in general prone to overestimation compared to GPS data. Despite such overestimation, UDs had high spatial congruence between proximity and GPS data for both home range (range: 0.84–0.91) and core areas (0.68–0.87). A high degree of overlapping indicates close juxtaposition of proximity‐ and GPS‐derived UDs in space. This can lead to reliable descriptions of spatial distributions of individuals within the sampling area using either location sampling method (Kochanny et al. 2009).

### 4.5 Conclusions

Our study demonstrates that proximity biologging systems, traditionally used for social interaction data, can effectively quantify the spatial patterns and utilization distributions (UDs) of small, free‐ranging animals. While the fixed detection grid constraints the absolute UDs size, the system reliably characterizes individual space use, site fidelity, and kinship‐driven overlap.

Our field simulations confirmed high spatial congruence between proximity‐ and GPS‐derived UDs, validating the system's accuracy. However, successful implementation requires accounting for (1) the system's capabilities in terms of detection range and autonomy, (2) environmental factors like vegetation density and its impact on system performance, and (3) the species biology being monitored. In our study, the utilization distributions are constrained by the area covered by the detection grid. This factor is closely related to the detection range of the SLs, as this determines the density of the detection grid in order to avoid blind spots in the monitored area. Increasing the resolution of utilization distribution estimations can be achieved by reducing the spacing between logging stations or increasing the number of SLs. However, as BLE signal penetration is sensitive to environmental factors such as vegetation density and humidity, in situ equipment calibration is essential for optimizing equipment deployment and the monitoring strategy. Further technical optimisations, such as increasing the detection range or signal penetration, would likely extend the maximum monitoring range, enabling a wider range of species to be monitored.

While our results demonstrate the high locational accuracy of IoT proximity loggers, the applicability of this technology is also bounded by the characteristics of the target species. Currently, this technology is most applicable to species with high site fidelity and area‐restricted movement patterns, as fast moving or wide‐ranging species might bypass detection by SLs. For example, the Spix's disk‐winged bat ( *Thyroptera tricolor* ) roosts consistently in the same area and a colony has a mean home range of less than one hectare (Chaverri and Kunz 2011; Vonhof et al. 2004). However, this method may not be suitable for species with a body mass of less than 11 g, such as *T. tricolor*, unless electronic components can be further miniaturized to reduce tag weight. Beyond bats, this methodology is applicable to other less vagile mammals, including rodents and lagomorphs (Broekman et al. 2023; Huels et al. 2025). In conclusion, IoT‐based proximity logging offers a robust, automated alternative for movement ecology studies where GPS is impractical or traditional methods are too labour‐intensive, particularly for identifying activity hotspots in conservation priority areas.

## Author Contributions

**Jesús R. Hernández‐Montero:** conceptualization (lead), data curation (lead), formal analysis (lead), investigation (lead), methodology (lead), visualization (supporting), writing – original draft (lead), writing – review and editing (equal). **Janis Maximilian Wolf:** data curation (supporting), formal analysis (supporting), methodology (supporting), visualization (lead), writing – review and editing (equal). **Fernanda Chávez:** methodology (supporting), writing – review and editing (equal). **Frieder Mayer:** funding acquisition (lead), project administration (lead), resources (lead), supervision (supporting), writing – review and editing (equal). **Gerald Kerth:** funding acquisition (lead), project administration (lead), resources (lead), supervision (lead), writing – review and editing (equal).

## Funding

This work was supported by German Research Fundation (DFG) (Grant KE746/16‐1).

## Conflicts of Interest

The authors declare no conflicts of interest.

## Supporting information


**Appendix S1:** Utilization distributions of free‐ranging bats and their overlap.
**Appendix S2:** Experiment utility distributions using proximity and GPS data.

## Data Availability

Data, scripts and analysis routines are available via the Open Science Framework repository https://osf.io/sg6dz/overview?view_only=4624cf1757b34e87bf7a4aca9d889319.
