# Supplementary material for: Determining Activity Patterns and Home Range of Wild Bats Using a Proximity Biologging System Based on the Internet of Things (IoT)
Source: Ecol Evol. 2026 May 11;16(5):e73604. doi: 10.1002/ece3.73604 (PMC13158582; doi:10.1002/ece3.73604)
Supplement: Supplementary file 1 — Appendix S1: Utilization distributions of free‐ranging bats and their overlap. Appendix S2: Experiment utility distributions using proximity and GPS data. [file ECE3-16-e73604-s001.zip › Appendix_1.pdf]

Determining activity patterns and home range of wild bats using a proximity biologging system based on the Internet of Things (IoT)

Appendix 1: Utilization distributions of free-ranging bats and their overlap

Jesús R. Hernández-Montero Janis M. Wolf Fernanda Chávez Frieder Mayer
Gerald Kerth

Table of contents

1 Introduction **3**

2 Methods **3**

3 Results - UD overlap 2024 and 2025 **4**
3.1 May 2024 - May24 4
3.1.1 Map - May24 5
3.1.2 Home Rage - May24 6
3.1.3 Core Area - May24 6
3.2 August 2024 - Aug24a 7
3.2.1 Map 1 - Aug24a 8
3.2.2 Map 2 - Aug24a 8
3.2.3 Home Range - Aug24a 9
3.2.4 Core Area - Aug24a 9
3.3 August 2024 - Aug24b 10
3.3.1 Map - Aug24b 10
3.3.2 Home Range - Aug24b 11
3.3.3 Core Area -Aug24b 11
3.4 May 2025 - May25 12
3.4.1 Map - May25 12
3.4.2 Home Range - May25 13
3.4.3 Core Area - May25 13
3.5 August 2025 - Aug25 14
3.5.1 Map - Aug25 14
3.5.2 Home Range 15
3.5.3 Core Area 15

4 Site fidelity - Same year **16**
4.0.1 Fidelity 2024 16
4.0.2 Fidelity 2025 16
4.0.3 UD overlap - Same year 17

5 Site fidelity - Different years **18**
5.0.1 UD overlap - Different year 22
5.0.2 HR area boxplot 23
5.0.3 CA area boxplot 23

6 Site Fidelity: UDs' area comparison **25**

6.1 Home range area comparison . . . 26
6.1.1 LMM summary . . . 26
6.1.2 HR area change . . . 26
6.2 Core area comparison . . . 27
6.2.1 LMM summary . . . 27
6.2.2 CA area change . . . 27

7 Utility distribution overlap **28**
7.1 Home Range Overlap - Kinship . . . 28
7.1.1 GLMM - HR Summary . . . 29
7.1.2 Boxplot - HR overlap by kinship . . . 30
7.2 Core Area Overlap - Kinship . . . 31
7.2.1 GLMM - CA Summary . . . 31
7.2.2 Boxplot - CA overlap by kinship . . . 32

1 Introduction

We calculate the utilization distribution of free-ranging bats using location data collected with a proximity biologging system based on the IoT: ProxLogs (Kirkpatrick et al. 2021). We marked bats with mobile loggers (ML) and monitored their activity within a detection grid installed at their core roosting area. The detection grid consisted of 65 stationary loggers (SL). The encounters between ML and SL were accounted as fixes to estimate individual home range and core areas. We assess pairwise home range and core area overlap between individuals using the utilization distribution overlapping index (UDOI) proposed by Fieberg and Kochanny (2005).

2 Methods

Fieldwork was carried out in the months of May and August in 2024 and 2025. We tagged 25 different bats in 2024 and 10 in 2025. However, given tag lost or potential malfunction we retrieved data from 21 different individuals marked in 5 different batches. Utilization distributions were calculated with the location data retrieved from each SL. We only considered those individuals that delivered at least 2 nights of data.

We analyzed the UDs overlap of **111 dyads with 103 unique dyads** sampled in 5 batches. The table below shows the details of each batch.

Table 1: Bats tagged in each sampling batch

Batch	No. bats (with info)	RFIDs sampled	No. dyads	Start date	End date
May24	8 (7)	[0A62, 418B, 663E, 922C, A24D, A42D, AC37]	21	2024-05-15	2024-05-17
Aug24a	12 (11)	[DFAD, 494F, 418B, B484, 2D31, 8B80, 0F3D, 22B0, 8C52, 04DB, 879F]	55	2024-08-12	2024-08-14
Aug24b	6 (5)	[5939, BF04, A24D, 076C, 1055]	10	2024-08-15	2024-08-17
May25	8 (5)	[076C, 418B, 494F, 922C, B484]	10	2025-05-14	2025-05-16
Aug25	6 (6)	[04DB, 22B0, 076C, 922C, 1055, AC37]	15	2025-08-07	2025-08-09

Sampling times

The time window used affects the size of the calculated UD. To avoid inflated overlapping between individuals given activity around dayroosts (*e.g.*, roost entrance or leaving) we decide to analyzed the data two hour after sunset and 3 hours before sunrise. Sampling times in May and August cover the following hours:

- May: 23:00 - 02:30 (210 minutes)
- August: 22:45 - 03:00 (255 minutes)

3 Results - UD overlap 2024 and 2025

3.1 May 2024 - May24

In May 2024, we tagged eight bats and they were monitored between the 15th and 17th. There were no mother-daughter pair tagged but `'0A62'` and `'A42D'` are sisters (mother: `'AA3C'`).

The following summary table presents the amount of data collected by each individual during the sampling period. The able shows the following columns

- `rfid`: identity of the individual according to its RFID tag
- `nights`: number of nights that each bat delivered proximity data
- `records`: number of signals recoreded by the stationary nodes
- `time_observed`: number of minutes with at least one record for bats moniotored in May 210 minutes, for bats monitored in August 255 minutes
- `time_expected`: number of minutes expected for the number of nights with data
- `pct_obs`: percentage of time a given bat was observed (i.e. detected)
- `batch`: name of the group of bats tagged together in the same sampling period

Table 2: Summary of the data collected by each individual in the sampling period for calculating utility distributions. The column 'pct_obs' indicates the percentage of time that the bat was detected by at least one statinary logger of the detection grid.

rfid	nights	records	time_observed	time_expected	pct_obs	batch
663E	2	3758	329	420	78	May24
922C	2	3648	304	420	72	May24
AC37	3	2314	415	630	66	May24
A42D	3	1520	319	630	51	May24
418B	2	1527	211	420	50	May24
A24D	2	950	154	420	37	May24
0A62	2	318	82	420	20	May24
4ECA	2	86	18	420	4	May24

As we can see, the bat `'4ECA'` was detected just 4% of the total expected time inside the detection grid during two nights. We decided to remove this bat from the analysis given its low amount of data.

3.1.1 Map - May24

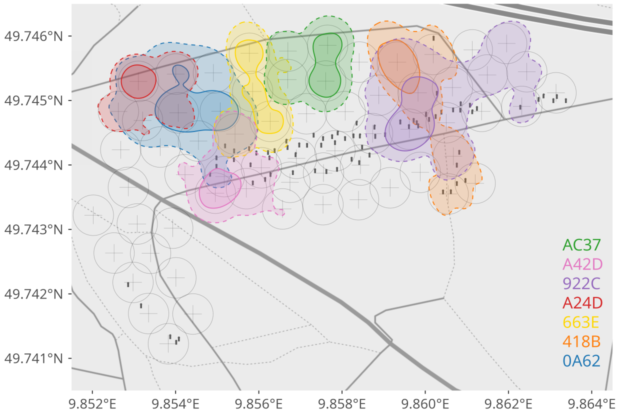

Figure 1: Home range (AKDE 95%, dashed lines) and core areas (AKDE 50%, solid lines) of bats marked in May 2024. In this figure and the following, crosses indicate the location of stationary loggers, circles around them indicate the estimated 35 m detection range. Black symbols represent bat boxes.

3.1.2 Home Rage - May24

Table 3: Home range overlap based on UDOI. Values on the diagonal are individual home range areas (ha)

	0A62	418B	663E	922C	A24D	A42D	AC37
0A62	4.115	NA	NA	NA	NA	NA	NA
418B	0.000	2.943	NA	NA	NA	NA	NA
663E	0.021	0.000	2.217	NA	NA	NA	NA
922C	0.000	0.382	0.000	4.895	NA	NA	NA
A24D	0.279	0.000	0.000	0.000	1.846	NA	NA
A42D	0.020	0.000	0.009	0.000	0.000	2.131	NA
AC37	0.000	0.000	0.002	0.000	0.000	0.000	2.019

3.1.3 Core Area - May24

Table 4: Core area overlap based on UDOI. Values on the diagonal are individual core areas (ha)

	0A62	418B	663E	922C	A24D	A42D	AC37
0A62	0.959	NA	NA	NA	NA	NA	NA
418B	0.000	0.470	NA	NA	NA	NA	NA
663E	0.000	0.000	0.575	NA	NA	NA	NA
922C	0.000	0.006	0.000	0.803	NA	NA	NA
A24D	0.000	0.000	0.000	0.000	0.268	NA	NA
A42D	0.000	0.000	0.000	0.000	0.000	0.366	NA
AC37	0.000	0.000	0.000	0.000	0.000	0.000	0.474

May 2024 remarks

- 8 bats were marked; however, 7 bats retrieved enough data making 21 possible dyads.
- Overlap is based on the UDOI index.
- **Home range**
  - Area (mean ± SD): 2.881 ± 1.183 ha.
  - Overlap (mean ± SD): 0.034 ± 0.1.
- **Core area**
  - Area (mean ± SD): 0.559 ± 0.244 ha.
  - Overlap (mean ± SD): 0 ± 0.001.

3.2 August 2024 - Aug24a

In August two batches of bats delivered proximity data. The first batch consisted of 12 bats monitored between the 12th and 14th of August. The following summary table shows the amount of data collected for each individual.

Table 5: Summary of the data collected by each individual in the sampling period for calculating utility distributions. The column 'pct_obs' indicates the percentage of time that the bat was detected by at least one statinary logger of the detection grid.

rfid	nights	records	time_observed	time_expected	pct_obs	batch
04D3	1	869	173	255	68	Aug2_24
0F3D	3	2946	500	765	65	Aug2_24
494F	3	3121	473	765	62	Aug2_24
DFAD	3	1664	394	765	52	Aug2_24
8B80	3	2139	393	765	51	Aug2_24
04DB	2	623	164	510	32	Aug2_24
2D31	3	941	241	765	32	Aug2_24
B484	3	746	231	765	30	Aug2_24
879F	2	553	127	510	25	Aug2_24
8C52	3	321	151	765	20	Aug2_24
22B0	3	311	143	765	19	Aug2_24
418B	3	283	128	765	17	Aug2_24

The bat `'04D3'` was detected only during 1 day and hence we removed it form the analysis. Given that our AKDE function can only map 8 individuals, we present two maps one of 6 and another of 5 individuals. The overlapping matrix of HR and CA present all the individuals together.

3.2.1 Map 1 - Aug24a

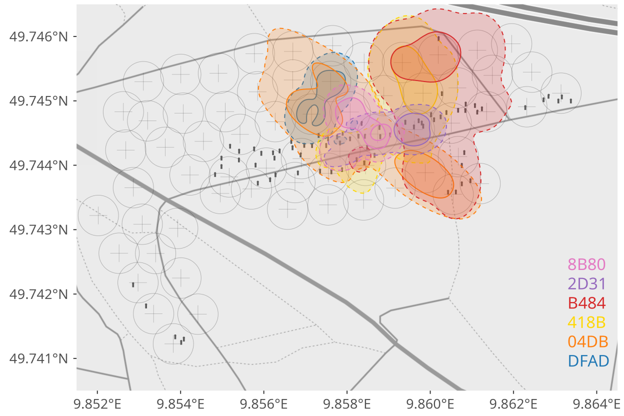

Figure 2: Home range (AKDE 95%, dashed lines) and core areas (AKDE 50%, solid lines) of bats marked in the second batch of bats in August 2024.

3.2.2 Map 2 - Aug24a

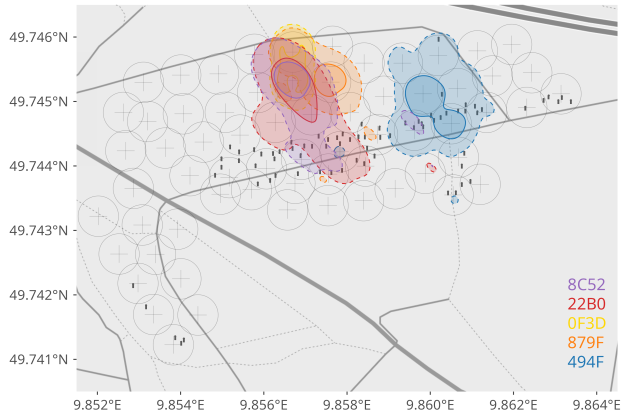

Figure 3: Home range (AKDE 95%, dashed lines) and core areas (AKDE 50%, solid lines) of bats marked in the second batch of bats in August 2024.

3.2.3 Home Range - Aug24a

Table 6: Home range overlap based on UDOI. Values on the diagonal are individual home range areas (ha)

	04DB	0F3D	22B0	2D31	418B	494F	879F	8B80	8C52	B484	DFAD
04DB	5.565	NA	NA	NA	NA	NA	NA	NA	NA	NA	NA
0F3D	0.131	0.987	NA	NA	NA	NA	NA	NA	NA	NA	NA
22B0	0.526	0.567	2.913	NA	NA	NA	NA	NA	NA	NA	NA
2D31	0.053	0.000	0.003	1.453	NA	NA	NA	NA	NA	NA	NA
418B	0.012	0.000	0.004	0.210	3.886	NA	NA	NA	NA	NA	NA
494F	0.002	0.000	0.000	0.208	0.809	2.861	NA	NA	NA	NA	NA
879F	0.405	0.115	0.116	0.000	0.000	0.000	1.577	NA	NA	NA	NA
8B80	0.233	0.000	0.022	0.232	0.026	0.001	0.025	1.548	NA	NA	NA
8C52	0.359	0.889	1.624	0.001	0.000	0.000	0.179	0.002	2.257	NA	NA
B484	0.025	0.000	0.000	0.020	0.838	0.447	0.000	0.000	0.000	6.104	NA
DFAD	0.459	0.007	0.307	0.000	0.000	0.000	0.766	0.016	0.190	0.000	1.363

3.2.4 Core Area - Aug24a

Table 7: Core area overlap based on UDOI. Values on the diagonal are individual core areas (ha)

	04DB	0F3D	22B0	2D31	418B	494F	879F	8B80	8C52	B484	DFAD
04DB	1.352	NA	NA	NA	NA	NA	NA	NA	NA	NA	NA
0F3D	0.000	0.276	NA	NA	NA	NA	NA	NA	NA	NA	NA
22B0	0.049	0.332	0.563	NA	NA	NA	NA	NA	NA	NA	NA
2D31	0.000	0.000	0.000	0.282	NA	NA	NA	NA	NA	NA	NA
418B	0.000	0.000	0.000	0.000	0.623	NA	NA	NA	NA	NA	NA
494F	0.000	0.000	0.000	0.000	0.314	0.610	NA	NA	NA	NA	NA
879F	0.097	0.000	0.000	0.000	0.000	0.000	0.236	NA	NA	NA	NA
8B80	0.001	0.000	0.000	0.000	0.000	0.000	0.000	0.383	NA	NA	NA
8C52	0.001	0.456	0.602	0.000	0.000	0.000	0.000	0.000	0.289	NA	NA
B484	0.000	0.000	0.000	0.000	0.152	0.001	0.000	0.000	0.000	0.778	NA
DFAD	0.223	0.000	0.016	0.000	0.000	0.000	0.461	0.000	0.000	0.000	0.349

August 2024 (Aug24a) remarks

- 12 bats were marked and retrieved data from 11 bats making 55 possible dyads.
- Overlap is based on the UDOI index.
- **Home range**
  - Area (mean ± SD): 2.774 ± 1.74 ha.
  - Overlap (mean ± SD): 0.179 ± 0.314.
- **Core area**
  - Area (mean ± SD): 0.559 ± 0.244 ha.
  - Overlap (mean ± SD): 0.049 ± 0.132.

3.3 August 2024 - Aug24b

The second batch of bats tagged in August consisted of 6 bats; however, five bats returned suitable data for analysis making 10 possible dyads. The following summary table shows the amount of data collected from each individual.

Table 8: Summary of the data collected by each individual in the sampling period for calculating utility distributions. The column 'pct_obs' indicates the percentage of time that the bat was detected by at least one statinary logger of the detection grid.

rfid	nights	records	time_observed	time_expected	pct_obs	batch
076C	3	3394	417	765	55	Aug3_24
1055	3	1102	352	765	46	Aug3_24
BF04	3	1172	336	765	44	Aug3_24
5939	3	756	217	765	28	Aug3_24
A24D	2	549	135	510	26	Aug3_24
EE51	1	135	49	255	19	Aug3_24

The individual `'EE51'` delivered only one night of data, therefore we removed from the UD overlap analysis.

3.3.1 Map - Aug24b

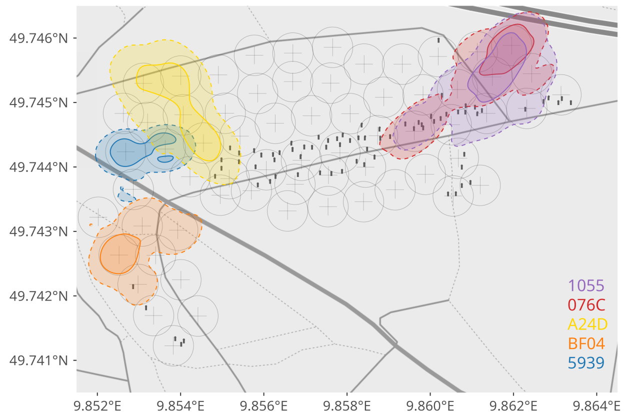

Figure 4: Home range (AKDE 95%, dashed lines) and core areas (AKDE 50%, solid lines) of bats marked in the third batch of bats in August 2024.

3.3.2 Home Range - Aug24b

Table 9: Home range overlap based on UDOI. Values on the diagonal are individual home range areas (ha)

	076C	1055	5939	A24D	BF04
076C	2.859	NA	NA	NA	NA
1055	1.247	3.633	NA	NA	NA
5939	0.000	0.000	1.272	NA	NA
A24D	0.000	0.000	0.013	3.355	NA
BF04	0.000	0.000	0.000	0.000	2.069

3.3.3 Core Area -Aug24b

Table 10: Core area overlap based on UDOI. Values on the diagonal are individual core areas (ha)

	076C	1055	5939	A24D	BF04
076C	0.516	NA	NA	NA	NA
1055	0.393	0.685	NA	NA	NA
5939	0.000	0.000	0.35	NA	NA
A24D	0.000	0.000	0.00	0.916	NA
BF04	0.000	0.000	0.00	0.000	0.362

August 2024 (2nd batch) remarks

- 6 bats were marked and retrieved data from 5 bats making 10 possible dyads.
- Overlap is based on the UDOI index.
- **Home range**
  - Area (mean ± SD): 2.638 ± 0.967 ha.
  - Overlap (mean ± SD): 0.126 ± 0.394.
- **Core area**
  - Area (mean ± SD): 0.566 ± 0.238 ha.
  - Overlap (mean ± SD): 0.039 ± 0.124.

3.4 May 2025 - May25

In May 2025, we tagged 8 bats and they were monitored between the 14th and 16th; however, only 5 bats provided sufficient data to analyze as shown in the following summary table.

Table 11: Summary of the data collected by each individual in the sampling period for calculating utility distributions. The column 'pct_obs' indicates the percentage of time that the bat was detected by at least one statinary logger of the detection grid.

rfid	nights	records	time_observed	time_expected	pct_obs	batch
494F	3	3136	353	630	56	May25
418B	2	1358	208	420	50	May25
04DB	1	291	89	210	42	May25
922C	2	1026	177	420	42	May25
076C	2	634	166	420	40	May25
B484	2	632	161	420	38	May25
1055	1	150	39	210	19	May25

The bats `'04DB'`, `'1055'`, and `'F1D5'` (not shown in the table) were recorded only during one night, hence they were removed from the analysis.

3.4.1 Map - May25

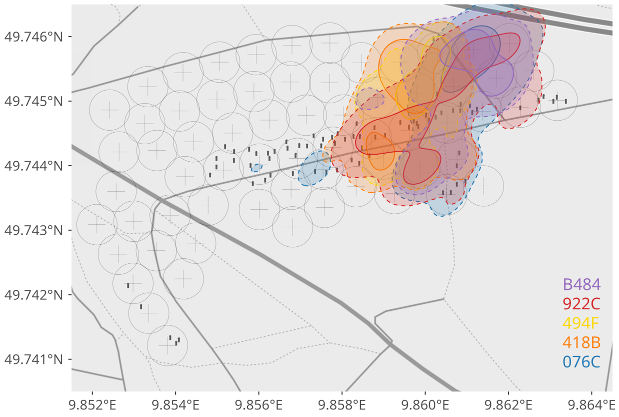

Figure 5: Home range (AKDE 95%, dashed lines) and core areas (AKDE 50%, solid lines) of bats marked in May 2025.

3.4.2 Home Range - May25

Table 12: Home range overlap based on UDOI. Values on the diagonal are individual home range areas (ha)

	076C	418B	494F	922C	B484
076C	5.687	NA	NA	NA	NA
418B	0.153	5.632	NA	NA	NA
494F	0.238	1.105	3.966	NA	NA
922C	0.951	0.473	0.465	7.073	NA
B484	1.586	0.194	0.272	0.674	4.774

3.4.3 Core Area - May25

Table 13: Core area overlap based on UDOI. Values on the diagonal are individual core areas (ha)

	076C	418B	494F	922C	B484
076C	0.755	NA	NA	NA	NA
418B	0.000	1.236	NA	NA	NA
494F	0.000	0.301	0.764	NA	NA
922C	0.223	0.012	0.006	1.833	NA
B484	0.432	0.000	0.002	0.081	0.841

May 2025 remarks

- 6 bats were marked and retrieved data from 5 making 10 possible dyads.
- Overlap is based on the UDOI index.
- **Home range**
  - Area (mean ± SD): 5.426 ± 1.16 ha.
  - Overlap (mean ± SD): 0.611 ± 0.47.
- **Core area**
  - Area (mean ± SD): 1.086 ± 0.462 ha.
  - Overlap (mean ± SD): 0.106 ± 0.157.

3.5 August 2025 - Aug25

In August 2025, we tagged 6 bats and they were monitored between the 7th and 9th. There were 15 possible dyads.

Table 14: Summary of the data collected by each individual in the sampling period for calculating utility distributions. The column 'pct_obs' indicates the percentage of time that the bat was detected by at least one statinary logger of the detection grid.

rfid	nights	records	time_observed	time_expected	pct_obs	batch
AC37	3	2395	442	765	58	Aug25
922C	2	2463	260	510	51	Aug25
04DB	2	1423	219	510	43	Aug25
076C	3	1652	323	765	42	Aug25
22B0	3	1516	240	765	31	Aug25
1055	2	673	94	510	18	Aug25

3.5.1 Map - Aug25

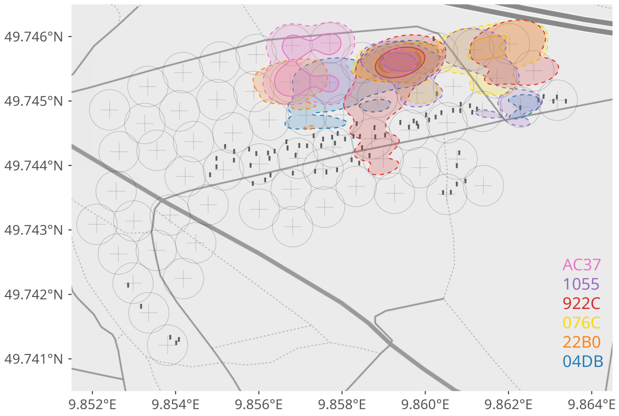

Figure 6: Home range (AKDE 95%, dashed lines) and core areas (AKDE 50%, solid lines) of bats marked in August 2025.

3.5.2 Home Range

Table 15: Home range overlap based on UDOI. Values on the diagonal are individual home range areas (ha)

	04DB	076C	1055	22B0	922C	AC37
04DB	2.448	NA	NA	NA	NA	NA
076C	0.818	2.762	NA	NA	NA	NA
1055	1.637	2.191	2.329	NA	NA	NA
22B0	2.084	0.912	1.421	1.603	NA	NA
922C	1.473	1.674	1.960	1.103	3.476	NA
AC37	0.228	0.020	0.039	0.267	0.027	2.169

3.5.3 Core Area

Table 16: Core area overlap based on UDOI. Values on the diagonal are individual core areas (ha)

	04DB	076C	1055	22B0	922C	AC37
04DB	0.224	NA	NA	NA	NA	NA
076C	0.703	0.477	NA	NA	NA	NA
1055	0.817	0.522	0.153	NA	NA	NA
22B0	0.772	0.497	0.756	0.145	NA	NA
922C	0.845	0.706	0.613	0.581	0.338	NA
AC37	0.000	0.000	0.000	0.000	0.000	0.692

August 2025 remarks

- 6 bats were marked and retrieved data from 6 making 15 possible dyads.
- Overlap is based on the UDOI index.
- **Home range**
  - Area (mean ± SD): 5.426 ± 1.16 ha.
  - Overlap (mean ± SD): 1.057 ± 0.79.
- **Core area**
  - Area (mean ± SD): 1.086 ± 0.462 ha.
  - Overlap (mean ± SD): 0.454 ± 0.346.

4 Site fidelity - Same year

Four bats were marked twice in the same year, two in 2024 (`['A24D', '418B']`) and two in 2025(`['076C', '922C']`). In this section we analyse the degree of site fidelity by addressing the overlap of their home range and corea calculated in each month (*i.e.,* May and August). We used the Bhattacharyya's affinity index to calculate UD overlap.

4.0.1 Fidelity 2024

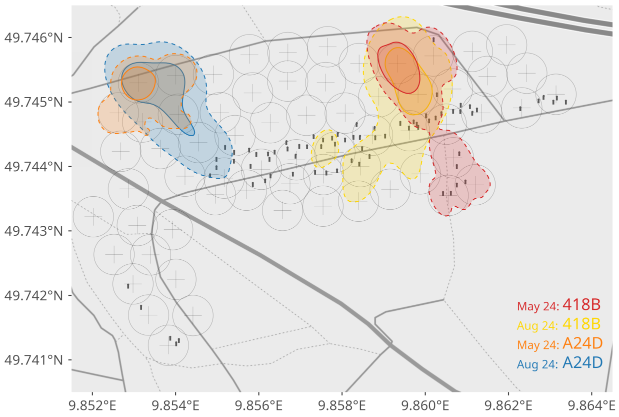

Figure 7: Fidelity of home range (AKDE 95%, dashed lines) and core areas (AKDE 50%, solid lines) of bats marked in May and August 2024.

4.0.2 Fidelity 2025

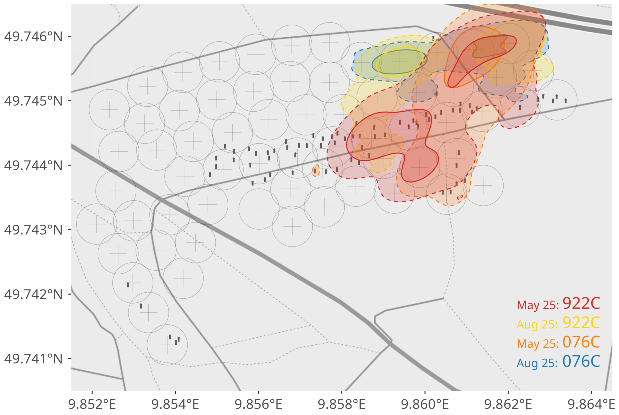

Figure 8: Home range (AKDE 95%, dashed lines) and core areas (AKDE 50%, solid lines) of bats marked in May and August 2025.

4.0.3 UD overlap - Same year

Table 17: Site fidelity. Overlap of home range and core areas of individuals tagged in May and August 2024 and 2025. Overlapping index based on BA

rfid	HR_overlap	HR_may	HR_aug	CA_overlap	CA_may	CA_aug
A24D	0.7256	1.772	3.111	0.5527	0.259	0.841
418B	0.8001	3.012	3.749	0.6564	0.461	0.610
076C	0.5442	5.190	2.815	0.2159	0.752	0.472
922C	0.2811	6.810	3.296	0.0000	1.681	0.313

Fidelity same year - remarks

- Four bats were re-tagged in the same year (May and August)
- The UD showed the following overlapping degree based on the BA index
  - Home range (mean ± SD): 0.588 ± 0.231
  - Core area (mean ± SD): 0.356 ± 0.303

5 Site fidelity - Different years

Nine individuals were tagged in both 2024 and 2025. The analysis of their UD can help us to infer if there is a site fidelity kept across years. We compare preferentially the same months when possible *e.g.*, May 2024 vs May 2025.

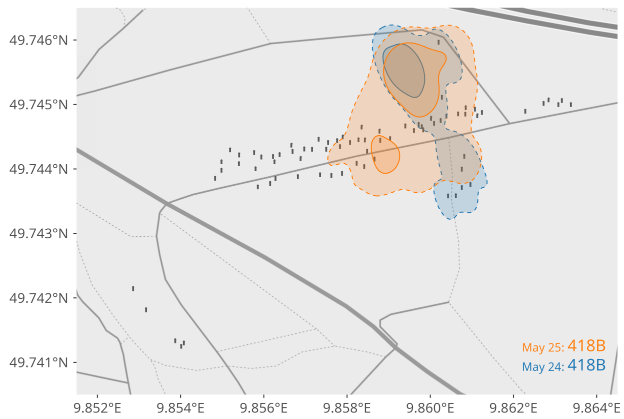

Figure 9: Home range (AKDE 95%, dashed lines) and core areas (AKDE 50%, solid lines) of bat 418B marked in May 2024 and 2025.

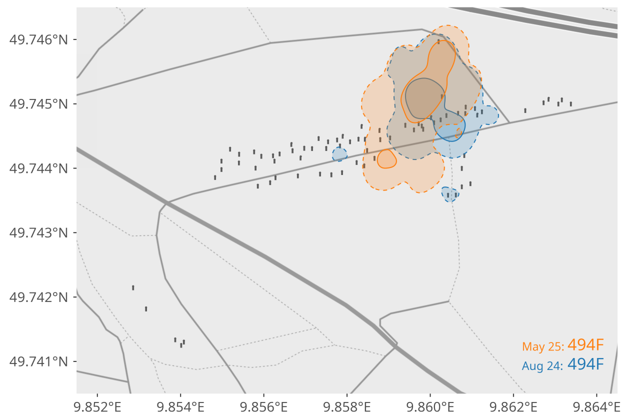

Figure 10: Home range (AKDE 95%, dashed lines) and core areas (AKDE 50%, solid lines) of bat 494F marked in August 2024 and May 2025.

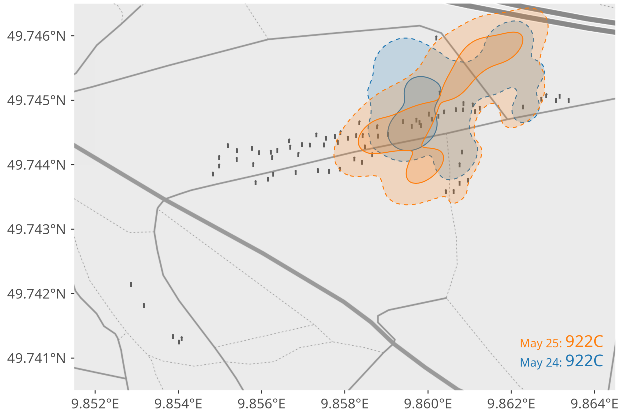

Figure 11: Home range (AKDE 95%, dashed lines) and core areas (AKDE 50%, solid lines) of bat 922C marked in May 2024 and 2025.

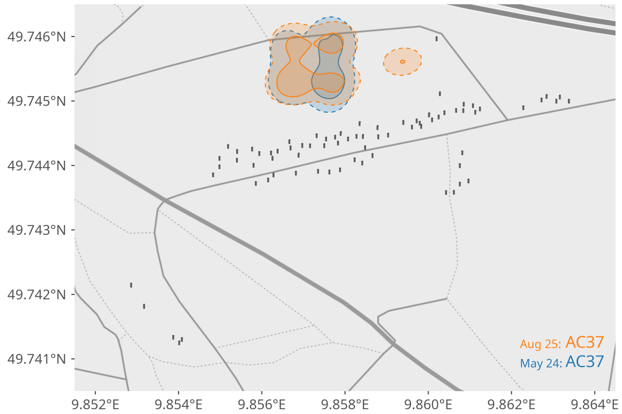

Figure 12: Home range (AKDE 95%, dashed lines) and core areas (AKDE 50%, solid lines) of bat AC37 marked in May 2024 and August 2025.

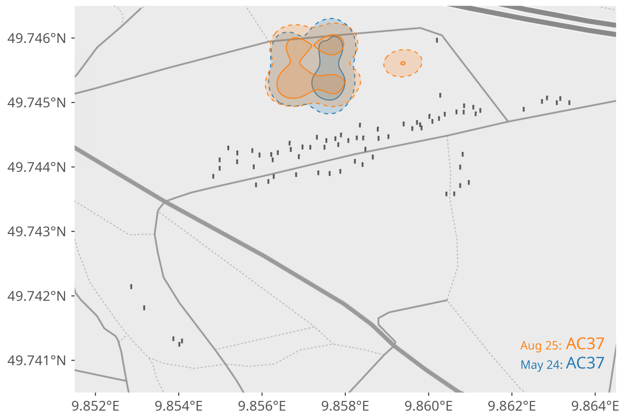

Figure 13: Home range (AKDE 95%, dashed lines) and core areas (AKDE 50%, solid lines) of bat 076C marked in August 2024 and May 2025.

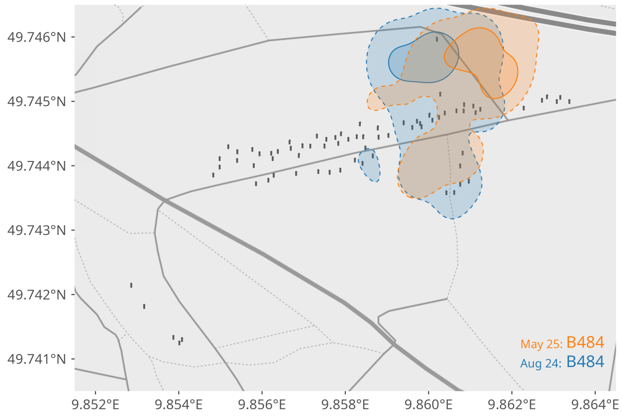

Figure 14: Home range (AKDE 95%, dashed lines) and core areas (AKDE 50%, solid lines) of bat B484 marked in August 2024 and May 2025. Black symbols represent bat boxes.

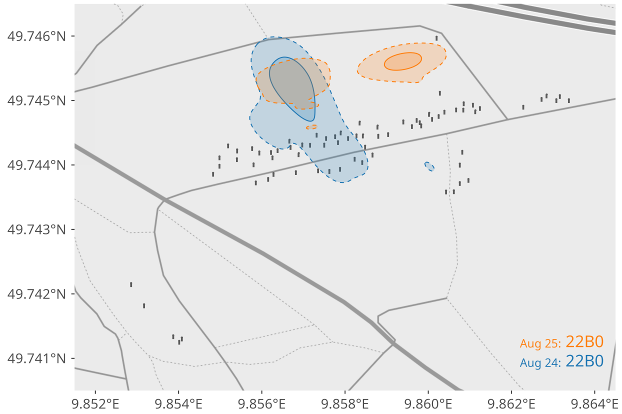

Figure 15: Home range (AKDE 95%, dashed lines) and core areas (AKDE 50%, solid lines) of bat 22B0 marked in August 2024 and 2025. Black symbols represent bat boxes.

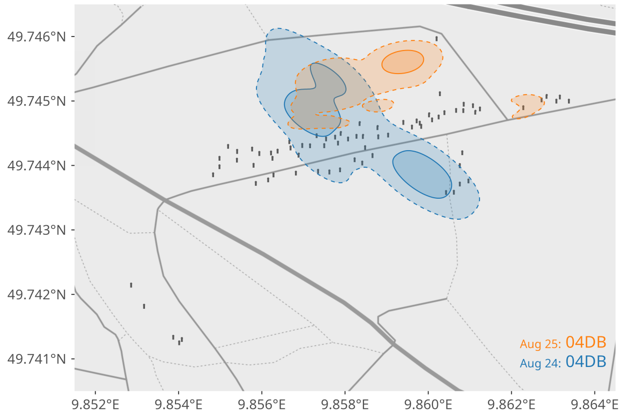

Figure 16: Home range (AKDE 95%, dashed lines) and core areas (AKDE 50%, solid lines) of bat 04DB marked in August 2024 and 2025. Black symbols represent bat boxes.

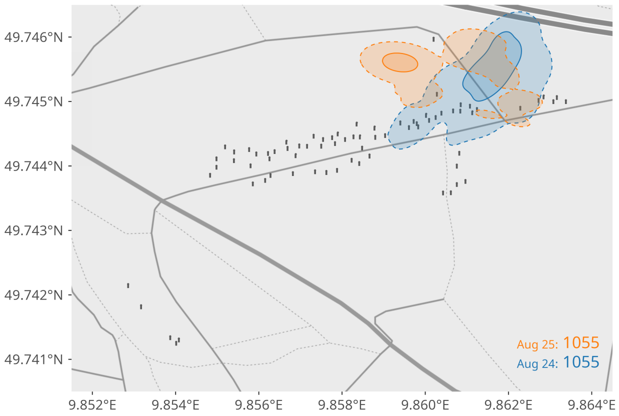

Figure 17: Home range (AKDE 95%, dashed lines) and core areas (AKDE 50%, solid lines) of bat 1055 marked in August 2024 and 2025. Black symbols represent bat boxes.

5.0.1 UD overlap - Different year

Table 18: Site fidelity. Overlap of home range and core areas of individuals tagged in 2024 and 2025. Overlapping index based on BA

rfid	HR_overlap	HR_24	HR_25	CA_overlap	CA_24	CA_25
418B	0.7364	2.943	5.632	0.6687	0.470	1.236
494F	0.7443	3.013	3.937	0.5874	0.612	0.812
922C	0.7384	4.895	7.073	0.4613	0.803	1.833
AC37	0.8724	1.960	2.249	0.4141	0.444	0.701
076C	0.7178	2.910	5.190	0.3130	0.530	0.752
B484	0.6108	6.186	5.182	0.0683	0.796	0.940
22B0	0.2842	2.913	1.603	0.0000	0.563	0.145
04DB	0.2612	5.565	2.448	0.0000	1.352	0.224
1055	0.1887	3.633	2.329	0.0000	0.685	0.153

5.0.2 HR area boxplot

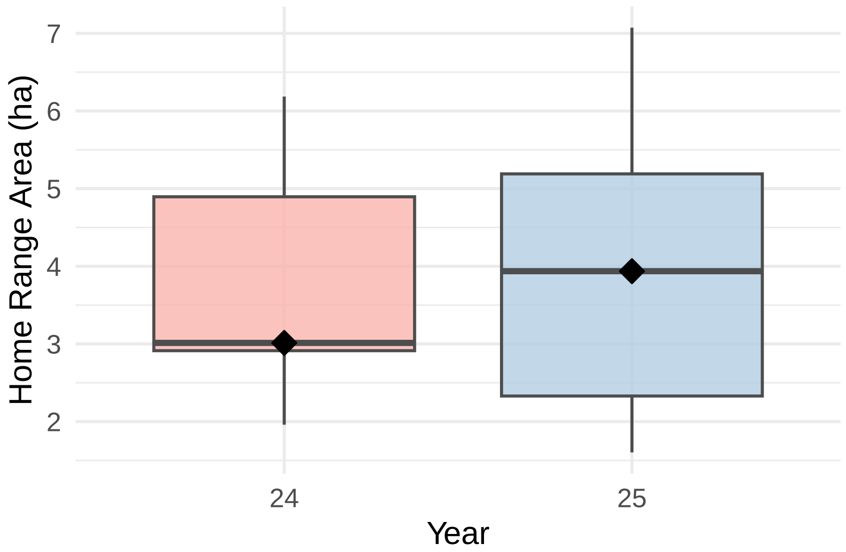

Figure 18: Home range area (ha) of retagged individuals in 2024 and 2025 (n = 9).

5.0.3 CA area boxplot

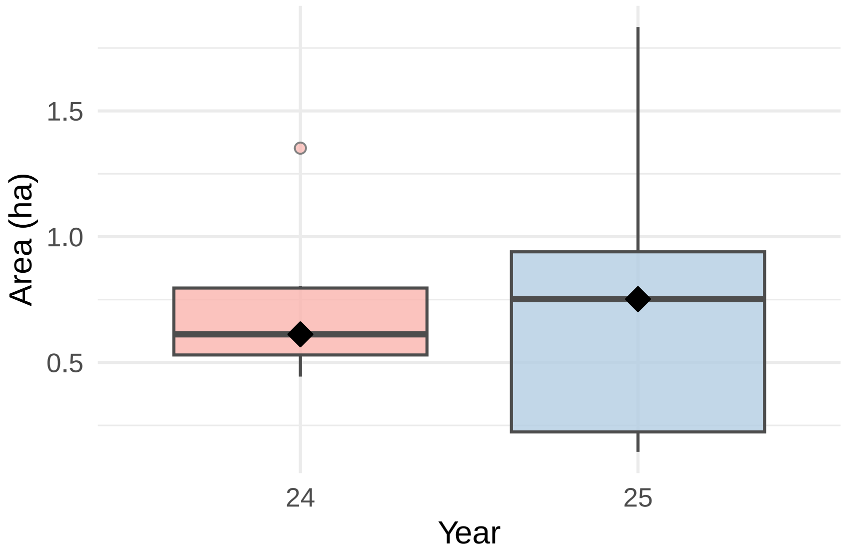

Figure 19: Core area (ha) of retagged individuals in 2024 and 2025 (n = 9).

Fidelity between years

- 9 bats delivered data in two different years
- Based on the BA index, individuals have a higher fidelity to their HR than to their CA
  - Home range (mean ± sd): 0.573 ± 0.256
  - Core area (mean ± sd): 0.279 ± 0.269
- All individuals showed a certain degree of overlap for their Home range
- 3 out of 9 individuals showed no overlap to their core area in different years
- The UD areas were not different from one year to another (HR: $p = 1$; CA: $p = 0.73$)

6 Site Fidelity: UDs' area comparison

We pool the data from repeatedly tagged bats to address for differences in the area between the first and second period of tagging. The first and second period were carried out either in the same year (n = 4) or in two different years (n = 9). Our test uses the data from 13 individuals as input, note that three individuals were repeatedly tagged in the same year and in different years. Since some individuals contribute in more than one trial to the dataset, we use Linear Mixed-Effects models (LMM) to account for the non-independence of these repeated measurements within the same individual.

The models takes the period of monitoring as a fix effect and individual ID ('`rfid'`) as a random effect, represented by the formula: `Area ~ Period + (1|rfid)`

Table 19: Information of utilization distributions areas(ha) and overlap based on the BA index of repeateadly tagged bats in the same and different years

rfid	HR_overlap	HR_first	HR_second	CA_overlap	CA_first	CA_second	Year
A24D	0.7256	1.772	3.111	0.5527	0.259	0.841	same
418B	0.8001	3.012	3.749	0.6564	0.461	0.610	same
076C	0.5442	5.190	2.815	0.2159	0.752	0.472	same
922C	0.2811	6.810	3.296	0.0000	1.681	0.313	same
B484	0.6108	6.186	5.182	0.0683	0.796	0.940	different
1055	0.1887	3.633	2.329	0.0000	0.685	0.153	different
AC37	0.8724	1.960	2.249	0.4141	0.444	0.701	different
418B	0.7364	2.943	5.632	0.6687	0.470	1.236	different
922C	0.7384	4.895	7.073	0.4613	0.803	1.833	different
22B0	0.2842	2.913	1.603	0.0000	0.563	0.145	different
076C	0.7178	2.910	5.190	0.3130	0.530	0.752	different
494F	0.7443	3.013	3.937	0.5874	0.612	0.812	different
04DB	0.2612	5.565	2.448	0.0000	1.352	0.224	different

6.1 Home range area comparison

6.1.1 LMM summary

```
Linear mixed model fit by REML. t-tests use Satterthwaite's method [
lmerModLmerTest]
Formula: area ~ period + (1 | rfid)
   Data: hr_area

REML criterion at convergence: 94.6

Scaled residuals:
    Min      1Q  Median      3Q     Max
-1.0852 -0.7350 -0.0945  0.7755  1.6907

Random effects:
 Groups   Name        Variance Std.Dev.
 rfid     (Intercept) 0.7985   0.8936
 Residual             1.8514   1.3606
Number of obs: 26, groups:  rfid, 10

Fixed effects:
             Estimate Std. Error      df t value Pr(>|t|)
(Intercept)    3.7991     0.4759 16.5816   7.982 4.47e-07 ***
periodsecond  -0.1683     0.5337 15.1503  -0.315    0.757
---
Signif. codes:  0 '***' 0.001 '**' 0.01 '*' 0.05 '.' 0.1 ' ' 1

Correlation of Fixed Effects:
            (Intr)
periodsecnd -0.561
```

6.1.2 HR area change

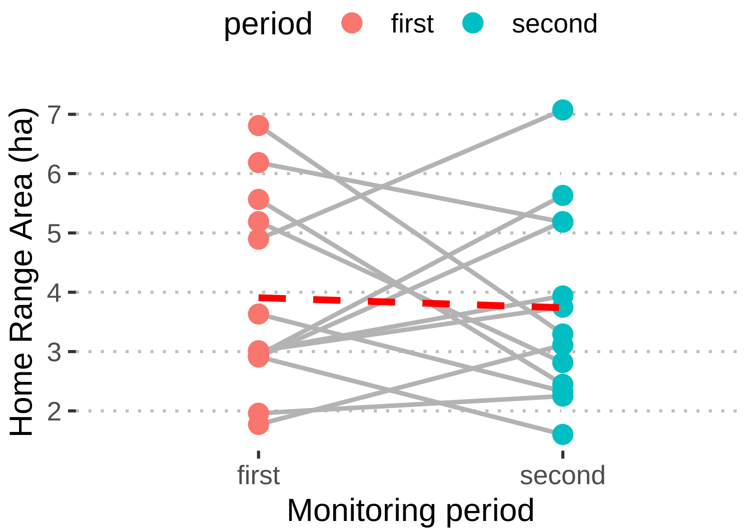

Figure 20: Individual trajectories of home range area. Gray lines show individual pairs. Red dashed line shows the mean trend.

6.2 Core area comparison

6.2.1 LMM summary

```
Linear mixed model fit by REML. t-tests use Satterthwaite's method [
lmerModLmerTest]
Formula: area ~ period + (1 | rfid)
   Data: ca_area

REML criterion at convergence: 33.2

Scaled residuals:
    Min      1Q  Median      3Q     Max
-1.2542 -0.5984 -0.1344  0.2452  2.5972

Random effects:
 Groups   Name        Variance Std.Dev.
 rfid     (Intercept) 0.001583 0.03979
 Residual             0.187445 0.43295
Number of obs: 26, groups:  rfid, 10

Fixed effects:
             Estimate Std. Error       df t value Pr(>|t|)
(Intercept)   0.72280    0.12082 22.02164   5.983 5.05e-06 ***
periodsecond -0.02892    0.16982 18.16615  -0.170    0.867
---
Signif. codes:  0 '***' 0.001 '**' 0.01 '*' 0.05 '.' 0.1 ' ' 1

Correlation of Fixed Effects:
            (Intr)
periodsecnd -0.703
```

6.2.2 CA area change

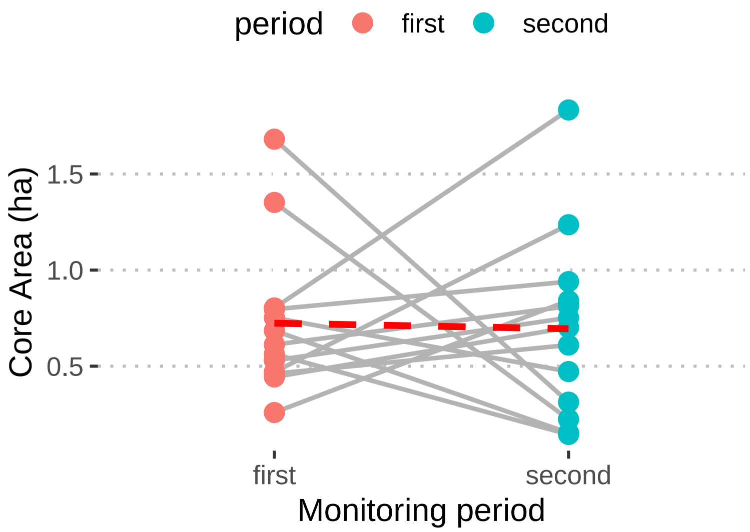

Figure 21: Individual trajectories of core area. Gray lines show individual pairs. Red dashed line shows the mean trend.

7 Utility distribution overlap

Below we show the general trends in home range and core area overlap considering 111 dyads across the 5 batches of bats. We considered overlapping values only from bats monitored in the same period.

In the following table we show the overlapping values for kin-related (n=9) and non-realted dyads (n=102)

Table 20: Utility distribution overlap (mean and SD) of kin and non-kin related dyads. Individuals under the category ‘same’ are retagged individuals

kinship	n	HR_ol_mean	HR_ol_sd	CA_ol_mean	CA_ol_sd
kin	9	0.79	0.74	0.21	0.24
non-kin	102	0.26	0.48	0.09	0.21
same	13	0.58	0.24	0.30	0.27

7.1 Home Range Overlap - Kinship

We assess siginificant differences in the overlapping degree of the HR between kin-related and non-related dyads. Additionally, we add to the input data the overlapping infomation of 13 individuals tracked in different times and categorized them as `'same'`. Because the number of dyads is not balanced between groups, we used a GLMM with Tweedie error distribution with log link. The response variable is the UDOI value predicted by kinship, we set the dyad identity as random factor to account for repeated measures, `'kin'` was set as the reference level of comparison (`UDOI ~ kinship + (1|dyad)`). The same analysis is done for the Core Area.

7.1.1 GLMM - HR Summary

```
 Family: tweedie  ( log )
Formula:          overlap_95 ~ kinship + (1 | dyad)
Dispersion:                  ~kinship
Data: overlap_kinship

      AIC       BIC    logLik -2*log(L)  df.resid
    127.6     143.8     -57.8     115.6       105

Random effects:

Conditional model:
 Groups Name        Variance Std.Dev.
 dyad   (Intercept) 0.5396   0.7345
Number of obs: 111, groups:  dyad, 103

Conditional model:
               Estimate Std. Error z value Pr(>|z|)
(Intercept)     -0.5657     0.4006  -1.412   0.1579
kinshipnon-kin  -1.0381     0.4526  -2.293   0.0218 *
---
Signif. codes:  0 '***' 0.001 '**' 0.01 '*' 0.05 '.' 0.1 ' ' 1

Dispersion model:
               Estimate Std. Error z value Pr(>|z|)
(Intercept)     -1.0915     0.7184  -1.520  0.12864
kinshipnon-kin   2.0064     0.6830   2.938  0.00331 **
---
Signif. codes:  0 '***' 0.001 '**' 0.01 '*' 0.05 '.' 0.1 ' ' 1
```

7.1.2 Boxplot - HR overlap by kinship

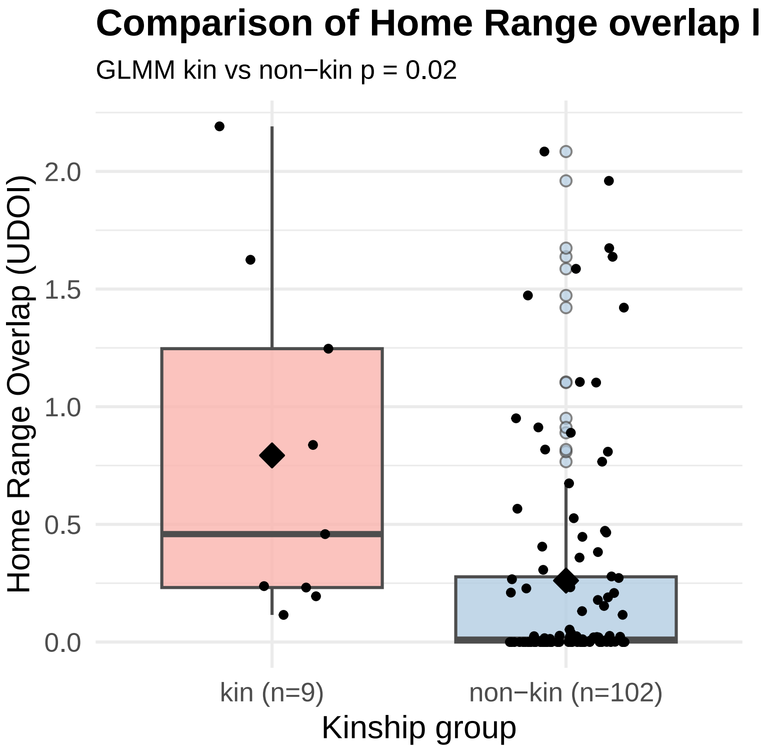

Figure 22: Home range overlap by kinship

7.2 Core Area Overlap - Kinship

7.2.1 GLMM - CA Summary

```
 Family: tweedie  ( log )
Formula:          overlap_50 ~ kinship + (1 | dyad)
Dispersion:                  ~kinship
Data: overlap_kinship

      AIC       BIC    logLik -2*log(L)  df.resid
    117.4     133.7     -52.7     105.4       105

Random effects:

Conditional model:
 Groups Name        Variance  Std.Dev.
 dyad   (Intercept) 1.698e-09 4.121e-05
Number of obs: 111, groups:  dyad, 103

Conditional model:
               Estimate Std. Error z value Pr(>|z|)
(Intercept)     -1.5592     0.5477  -2.847  0.00441 **
kinshipnon-kin  -0.8600     0.6331  -1.358  0.17433
---
Signif. codes:  0 '***' 0.001 '**' 0.01 '*' 0.05 '.' 0.1 ' ' 1

Dispersion model:
               Estimate Std. Error z value Pr(>|z|)
(Intercept)      0.5037     0.4379   1.150    0.250
kinshipnon-kin   1.0677     0.4251   2.511    0.012 *
---
Signif. codes:  0 '***' 0.001 '**' 0.01 '*' 0.05 '.' 0.1 ' ' 1
```

7.2.2 Boxplot - CA overlap by kinship

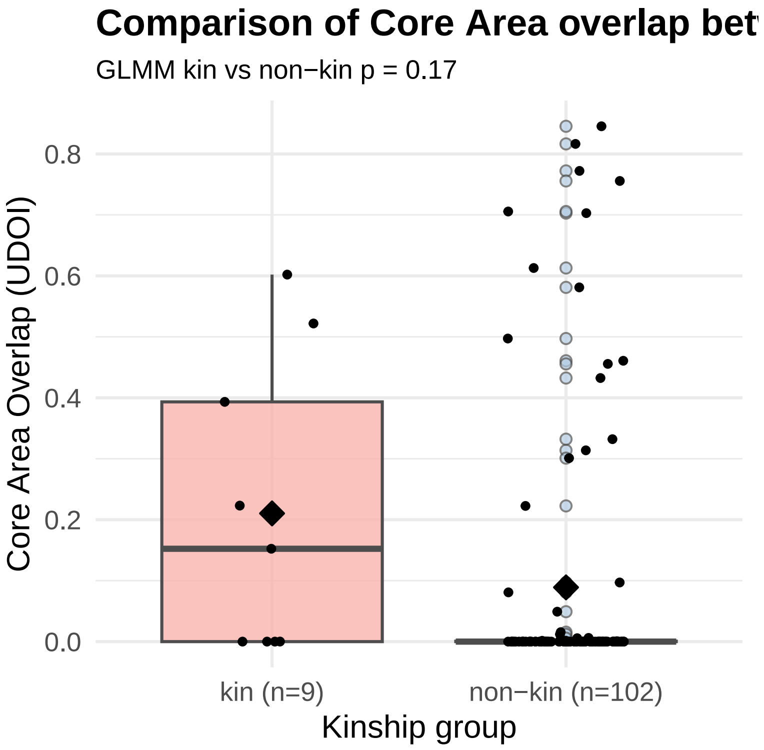

Figure 23: Core area overlap by kinship

UD areas and overlap

- 21 different bats were monitored with proximity loggers some bats were marked more than once
- We calculated the Home Range and Core Area areas and overlap based on 111 dyads
- **UD areas** (mean ± SD and range):
  - Home range: 3.111 ± 1.575 ha; range [0.987 - 7.073]
  - Core area: 0.587 ± 0.366 ha; range [0.145 - 1.833]
- **UD overlap** (mean ± SD and range):
  - Home range:0.333 ± 0.505; range [0 - 2.1914]
  - Core area: 0.12 ± 0.232; range [0 - 0.8454]
- Kin-related individuals have a significant higher degree of overlap in their HR (GLMM with Tweedie error distribution p = 0.02) than non-related pairs.
- The overlapping degree of the CA was not affected by kinship (GLMM with Tweedie error distribution p = 0.17).
