# Supplementary material for: Determining Activity Patterns and Home Range of Wild Bats Using a Proximity Biologging System Based on the Internet of Things (IoT)
Source: Ecol Evol. 2026 May 11;16(5):e73604. doi: 10.1002/ece3.73604 (PMC13158582; doi:10.1002/ece3.73604)
Supplement: Supplementary file 1 — Appendix S1: Utilization distributions of free‐ranging bats and their overlap. Appendix S2: Experiment utility distributions using proximity and GPS data. [file ECE3-16-e73604-s001.zip › Appendix_2.pdf]

Determining activity patterns and home range of wild bats using a proximity biologging system based on the Internet of Things (IoT)

Appendix 2: Experiment utility distributions using proximity and GPS data

Jesús R. Hernández-Montero Janis M. Wolf Fernanda Chávez Frieder Mayer Gerald Kerth

Table of contents

1 Introduction **2**

2 Methods **2**

2.1 Experimental set up 2

2.2 Data analysis 2

3 Results **4**

3.1 Utilitization distribution maps 5

3.2 Overlap of Utilization distributions 6

3.3 Utilization distribution comparisons 8

3.3.1 Home Range 8

3.3.2 Core Area 9

4 Calibration - SL detection range **10**

1 Introduction

Location data is the main raw input to estimate utility distributions (UD) of animals. In this notebook, we determine the home range (HR) and core area (CA) using auto-correlated kernel density estimation (AKDE) at 95% and 50% respectively. We compare the UDs derived from location data collected with proximity loggers (ProxLogs) and a GPS simultaneously during seven walks, three in May and four in August 2025. The area (ha) of each UDs is calculated, the percentage of over- or under-estimation is calculated taking the UDs derived from GPS data as reference. The degree of overlap is calculated with the Bhattacharyya's affinity index (BA).

2 Methods

2.1 Experimental set up

We collected data simultaneously from proximity mobile loggers (ML) and GPSs (OsmAnd v.5.0.5) during walks within the detection grid that we had set up in the core roosting area. Four persons took part in the experiment, each person carrying a ML and a GPS. MLs were mounted on 2 m high poles and were set with boosted transmission power at a sample rate of 2 s, the 65 stationary loggers (SL) from the detection grid were set in hidden mode. Each person tracked a 45-minute walk with a GPS set to take a waypoint every 2 seconds to match the sample rate of the MLs. Every person intentionally used a specific area of the forest to recreate an individual home range and core area. All participants started and ended at the same point, simulating the emergence and return of bats to a daytime roost.

2.2 Data analysis

Waypoints taking during the track of the walk as well as encounter between MLs and SNs were accounted as fixes for calculating UDs. Since each fix from the proximity data is linked to the location of a given SL, we randomly assigned the location of each data point according to its RSSI value. Since RSSI decreases with distance, records with an RSSI higher than -80 dB were placed within a 15-meter radius circle around the corresponding SL. Records with an RSSI lower than -80 dB were placed within a radius ranging from 15 to 35 meters. This random allocation reflects the detection range of the SL and avoids data points with identical location prior to home range estimation.

We calculated 95%-AKDEs to determine individual home ranges (HR), and 50%-AKDEs as core areas (CA, Samuel et al., 1985). Home range estimations via AKDE were computed using the R-package Animal Movement Tools (Signer et al., 2019, Signer et al., 2024). Utility distributions and its map projection is calculated with an R custom-made function **`cAKDE`** developed by Janis Wolf. The main arguments are shown below, to inspect all the argument, please refer to the R script **`func_cAKDE_amt_prox.R`**

```
# Function to calculate UD, main arguments are shown.

func_cAKDE(
    # a list of vectors per bat and sesion in the format
    # 'list(c("RIFD","start_date","end_date"))'
    rfid_batdays,
    # Starting time threshold to include in the analysis
    thresh_time_start,
    # Ending time threshold of the analysis
    thresh_time_end,
    # Whether the overlap should be calculated for each dyad
    overlap = TRUE,
    # Index for calculating overlap i.e. "udoi", "ba"
    ol_index = "udoi",
    # Other arguments
    ...
```

```
)
```

The functions returns two outputs. The first output is a map with the UD (home range and core area) of each individual included in the list passed to the function's argument `'rfid_batdays'`. The second output is a table with the areas and overlap degree, at HR and CA level, calculated for each dyad of individuals.

3 Results

In the following table we summarize the number of fixes recorded during each walk using proximity loggers and gps.

Table 1: Number of fixes recorded by proximity loggers (Prx) and GPS (gps)

Ind.	Prx	GPS
JW	611	555
LC	518	475
JM	990	781
CF	527	492
WJ	385	353
CL	376	354
MJ	303	284
Total	3710	3294

3.1 Utilitization distribution maps

The following figures show the utilization distributions derived from proximity and GPS data.

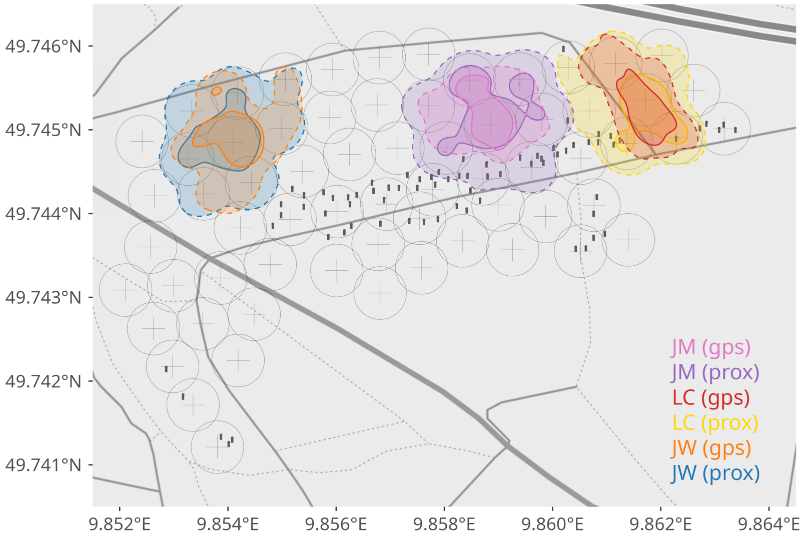

Figure 1: Home range (AKDE 95%, dashed lines) and core areas (AKDE 50%, solid lines) calculated from location data simultaneously collected with proximity loggers ("prox") and GPS ("gps") in May. Crosses indicate the location of stationary loggers, circles around them indicate the estimated 35 m detection range. Black symbols represent bat boxes.

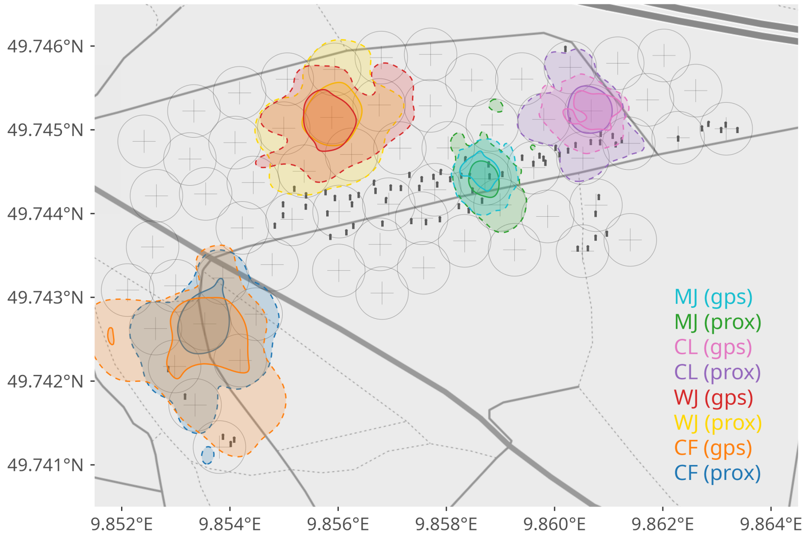

Figure 2: Home range (AKDE 95%, dashed lines) and core areas (AKDE 50%, solid lines) calculated from location data simultaneously collected with proximity loggers ("prox") and GPS ("gps") in August. Crosses indicate the location of stationary loggers, circles around them indicate the estimated 35 m detection range. Black symbols represent bat boxes.

3.2 Overlap of Utilization distributions

Table 2: Home range and core areas (ha) calculated with Proximity and GPS data. Overestimation percentage of the utility distributions is calculated taken GPS data as reference. Overlap degree is based on the Bhattacharyya's affinity index

Ind	HR_prox	HR_gps	HR_overest	HR_over-lap	CA_prox	CA_gps	CA_overest	CA_overlap
JW	2.887	2.080	38.80	0.8837	0.700	0.461	51.84	0.7893
LC	2.619	1.451	80.50	0.8798	0.583	0.424	37.50	0.7851
JM	3.326	1.661	100.24	0.8448	0.858	0.434	97.70	0.7837
CF	2.933	4.088	-28.25	0.8533	0.475	0.917	-48.20	0.7685
WJ	2.638	2.157	22.30	0.8932	0.490	0.418	17.22	0.8695
CL	1.685	0.898	87.64	0.8629	0.294	0.256	14.84	0.7802
MJ	0.916	0.672	36.31	0.9124	0.153	0.164	-6.71	0.6832

UDs overlap - Remarks

- We calculated home range (aKDE 95%) and core areas (aKDE 50%) using proximity and GPS data collected simultaneously during seven walks (three in May and four in August).
- Both UDs overlap in a high degree; however, UDs calculated with proximity data are larger (i.e. overestimated) than those calculated with GPS data.
- The mean ± SD and range values of UDs overestimation and overlap are given below:
- **Home range**:
  - Overestimation: 48.22 ± 44.82%. Range: [-28.25, 100.24]

- Overlap: 0.876 ± 0.024%. Range: [0.8448, 0.9124]

- **Core area**:
  - Overestimation: 23.46 ± 45.96%. Range: [-48.2, 97.7]
  - Overlap: 0.78 ± 0.054%. Range: [0.6832, 0.8695]

3.3 Utilization distribution comparisons

In this section we statistically compare the area of the UD derived from GPS and Proximity data (GPS vs ProxLogs).

3.3.1 Home Range

```
    Shapiro-Wilk normality test

data:  exp_overlap_all$HR_gps
W = 0.88351, p-value = 0.2425


    Shapiro-Wilk normality test

data:  exp_overlap_all$HR_prox
W = 0.8775, p-value = 0.2156


    Bartlett test of homogeneity of variances

data:  list(exp_overlap_all$HR_gps, exp_overlap_all$HR_prox)
Bartlett's K-squared = 0.49284, df = 1, p-value = 0.4827


    Paired t-test

data:  exp_overlap_all$HR_gps and exp_overlap_all$HR_prox
t = -1.698, df = 6, p-value = 0.1404
alternative hypothesis: true mean difference is not equal to 0
95 percent confidence interval:
 -1.3938617  0.2518617
sample estimates:
mean difference
         -0.571
```

3.3.2 Core Area

```
    Shapiro-Wilk normality test

data:  exp_overlap_all$CA_gps
W = 0.8404, p-value = 0.1002


    Shapiro-Wilk normality test

data:  exp_overlap_all$CA_prox
W = 0.98626, p-value = 0.9842


    Bartlett test of homogeneity of variances

data:  list(exp_overlap_all$CA_gps, exp_overlap_all$CA_prox)
Bartlett's K-squared = 3.6707e-06, df = 1, p-value = 0.9985


    Paired t-test

data:  exp_overlap_all$CA_gps and exp_overlap_all$CA_prox
t = -0.67509, df = 6, p-value = 0.5248
alternative hypothesis: true mean difference is not equal to 0
95 percent confidence interval:
 -0.3164510  0.1795939
sample estimates:
mean difference
    -0.06842857
```

4 Calibration - SL detection range

To determine the detection range of the Stationary Loggers (SL) in the field, we conduct a calibration experiment using two SLs and one Mobile Logger (ML). The signal strength as well as the sampling percentage were meassured at 5-meter steps over 2:50 minutes. With a sample rate of 2s we expected to have 85 records for each SL for a total of 170 records at each distance step in total. The following table shows the distance between ML and SLs at each step. Note that the ML move away from one SL and closer to a second SL.

Table 3: Distance in meters between Mobile Node and Stationary Loggers. Each time step last 2:50 for an expected number of records of 170 at each distance category between the two SL.

Time	ML-SN101	ML-SN102
1	65	0
2	60	5
3	55	10
4	50	15
5	45	20
6	40	25
7	35	30
8	30	35
9	25	40
10	20	45
11	15	50
12	10	55
13	5	60
14	0	65

The following summary table shows basic statistics of the signal strength and number of records at each distance category.

Table 4: Summary statistics of the signal strength (rssi), number of records and sampling percentage based on 170 expected records per distance category.

distance	median_rssi	mean_rssi	std_rssi	min_rssi	max_rssi	count_rssi	sampling_pct
0	-55	-55.97842	5.1632307	-89	-52	139	0.82
5	-77	-73.41611	5.9047629	-84	-65	149	0.88
10	-78	-76.73469	1.9700724	-80	-72	147	0.86
15	-81	-79.25949	3.9263610	-88	-74	158	0.93
20	-83	-82.32468	3.6116788	-89	-77	154	0.91
25	-87	-87.51807	1.8764357	-92	-83	83	0.49
30	-83	-83.39873	2.8237840	-89	-79	158	0.93
35	-89	-89.03846	1.5241971	-94	-86	78	0.46
40	-90	-89.80435	1.0026535	-91	-87	46	0.27
45	-89	-89.24444	1.3340907	-92	-87	45	0.26
50	-90	-90.64000	1.8457158	-95	-88	25	0.15
55	-91	-90.45455	0.9341987	-92	-89	11	0.06
60	-91	-91.00000	1.5118579	-94	-88	15	0.09
65	-90	-90.40000	0.8944272	-92	-90	5	0.03

The following plot shows the distribution of the signal strength and sampling percentage at each distance category.

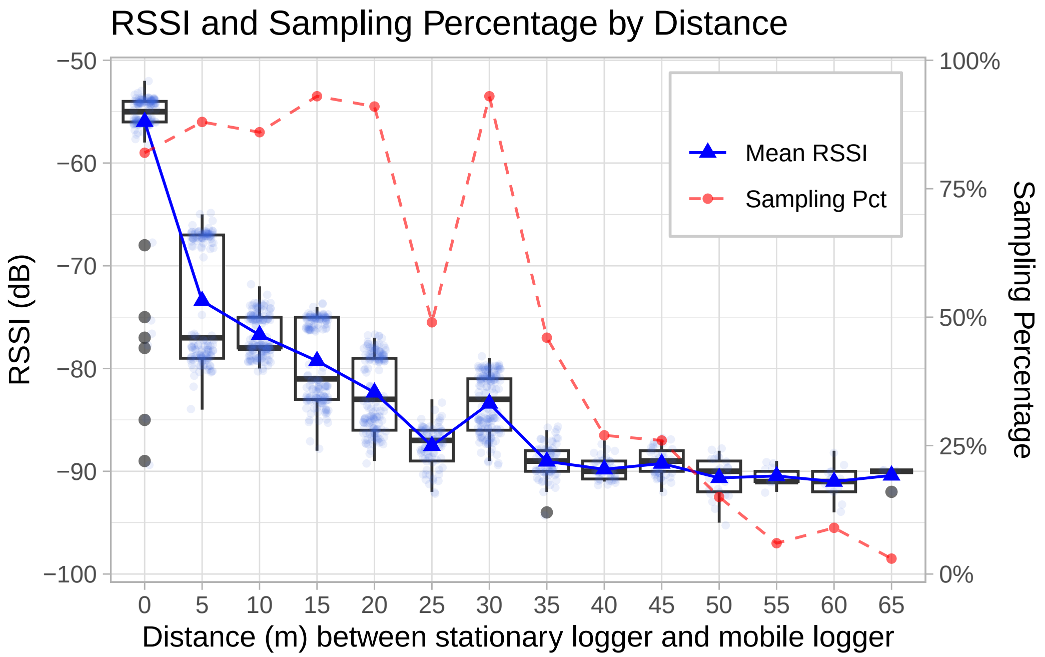

Figure 3: Signal strength (RSSI) distribution and sampling percentage over each distance category. The red dashed line represent the sampling percentage based on 170 expected records over 2:50 minutes at each distance step. The blue line represent the mean RSSI value at each distance. Note that the sampling percentage drops at 40-meters.

! Signal strength distribution

The sampling percentage drops below 46% after 35 metres. We consider records with values below -90 dB to be noise, as these are either from sources that are too far away from the logging station, or are bounced signals. Based on this information, we decided to space the SL at 65 metres to create a detection grid with a certain degree of overlap between neighbouring SLs.

References

Samuel, M. D., Pierce, D. J., & Garton, E. O. (1985). Identifying areas of concentrated use within the home range [Publisher: [Wiley, British Ecological Society]]. *Journal of Animal Ecology*, *54*(3), 711–719. https://doi.org/10.2307/4373

Signer, J., Fieberg, J., & Avgar, T. (2019). Animal movement tools (amt): R package for managing tracking data and conducting habitat selection analyses [_eprint: https://onlinelibrary.wiley.com/doi/pdf/10.1002/ece3.4823]. *Ecology and Evolution*, *9*(2), 880–890. https://doi.org/10.1002/ece3.4823

Signer, J., Smith, B., Reineking, B., Schlaegel, U., Fieberg, J., O'Brien, J., Niebuhr, B., Robitaille, A., Tal, A., & LaPoint, S. (2024, April 1). *Animal movement tools* [Institution: Comprehensive R Archive Network Pages: 0.2.2.0]. https://cran.r-project.org/web/packages/amt
